# A Link between Replicative Stress, Lamin Proteins, and Inflammation

**DOI:** 10.3390/genes12040552

**Published:** 2021-04-09

**Authors:** Simon Willaume, Emilie Rass, Paula Fontanilla-Ramirez, Angela Moussa, Paul Wanschoor, Pascale Bertrand

**Affiliations:** Université de Paris and Université Paris-Saclay, INSERM, iRCM/IBFJ, CEA, UMR Stabilité, Génétique Cellules Souches et Radiations, F-92265 Fontenay-aux-Roses, France; simon.willaume@cea.fr (S.W.); emilie.rass@cea.fr (E.R.); phdfontanilla@gmail.com (P.F.-R.); angela.moussa@hotmail.com (A.M.); paul.wanschoor@cea.fr (P.W.)

**Keywords:** DNA replication stress, lamins, Hutchinson-Gilford progeria syndrome, inflammation, senescence, aging, cancer, cGAS-STING pathway, double-strand break repair, genome instability

## Abstract

Double-stranded breaks (DSB), the most toxic DNA lesions, are either a consequence of cellular metabolism, programmed as in during V(D)J recombination, or induced by anti-tumoral therapies or accidental genotoxic exposure. One origin of DSB sources is replicative stress, a major source of genome instability, especially when the integrity of the replication forks is not properly guaranteed. To complete stalled replication, restarting the fork requires complex molecular mechanisms, such as protection, remodeling, and processing. Recently, a link has been made between DNA damage accumulation and inflammation. Indeed, defects in DNA repair or in replication can lead to the release of DNA fragments in the cytosol. The recognition of this self-DNA by DNA sensors leads to the production of inflammatory factors. This beneficial response activating an innate immune response and destruction of cells bearing DNA damage may be considered as a novel part of DNA damage response. However, upon accumulation of DNA damage, a chronic inflammatory cellular microenvironment may lead to inflammatory pathologies, aging, and progression of tumor cells. Progress in understanding the molecular mechanisms of DNA damage repair, replication stress, and cytosolic DNA production would allow to propose new therapeutical strategies against cancer or inflammatory diseases associated with aging. In this review, we describe the mechanisms involved in DSB repair, the replicative stress management, and its consequences. We also focus on new emerging links between key components of the nuclear envelope, the lamins, and DNA repair, management of replicative stress, and inflammation.

## 1. Introduction

DNA replication progression is constantly being challenged. Indeed, as a result of DNA lesions induced by both endogenous and exogenous agents, replication forks (RFs) can be slowed down or arrested. Moreover, inherent RF obstacles such as active transcription mechanism, tightly bound protein–DNA complexes, unusual DNA structures, and oncogene activation could also obstruct the progression of RFs. Replication stress is defined as the temporary slowing or stalling of RFs due to the encountered obstacles. In response to replication stress, mechanisms allow the DNA replication machinery to overcome DNA lesions or intrinsic RF obstacles. Therefore, replication stress response is crucial to safeguard the faithful transmission of genetic information to daughter cells [1,2].

Several mechanisms implicated in the response to replication stress have been identified. RFs are submitted to controlled remodeling and processing by the action of several specialized proteins to overcome DNA lesions [2]. To ensure the integrity of RFs during the process, forks are protected from nucleolytic degradation such as a potential (over)-resection that would have deleterious consequences on genomic stability [3]. Moreover, increasing evidence implicates DNA damage persistence and replication stress in the activation of immune responses. Indeed, the induction of replication stress and a defective DNA damage response (DDR) can lead to the presence of self-derived DNA in the cytoplasmic compartment [4,5]. Specialized cytoplasmic sensors detect the presence of DNA as a danger signal, thereby triggering the innate immune response. Although the mechanisms of DNA extrusion remain elusive, several studies point to a compromised nuclear structure as one of the main plausible reasons leading to the presence of endogenous DNA in the cytoplasm [6,7]. Of note, the rupture of the nuclear envelope (NE) has been associated with defects in the nuclear lamina (NL), a fibrous network lining the inner side of the NE. Nuclear lamins are proteins that participate in several crucial cellular processes widely beyond granting support and structure to the NE. Such a role of lamins is seen in DNA replication and DNA repair. Although the role of lamins in these processes requires further characterization, several studies propose that disruption in the integrity of nuclear lamins might hinder the recruitment of key factors of DNA replication and RF protection. Moreover, pathologies involving lamins alterations such as laminopathies are characterized by nuclear fragility and DNA damage accumulation, which in turn might contribute to strengthening the recently described DNA damage-induced inflammation [8]. Here, we review the mechanisms of the replication-stress response and their connection with the emerging crosstalk between replication stress and the immune response. Moreover, we highlight the role of nuclear integrity, namely the role of lamin proteins, which are the main components of the NL, in the maintenance of genome stability. Understanding the mechanisms that underlie genome stability and the innate immune response is crucial and may contribute to the development of therapy against cancer and aging.

## 2. Mechanisms of Double-Strand Break Repair

Different cellular mechanisms ensure genome stability upon DNA damage. Among the most toxic DNA lesions are double-strand breaks (DSBs). Programmed DSBs are induced during meiosis and V(D)J recombination to ensure genome variability or adaptability. Recently, genomic regions with recurrent DSBs in primary neural stem cells were identified, potentially generating neuronal diversity [9]. Moreover, induction of physiological DSB was observed in promoters of specific genes regulating their expression during neurogenesis [10]. DSBs also arise from endogenous cellular metabolism or can be induced upon exogenous genotoxic stresses such as in anti-cancer therapy or accidental exposure to genotoxic agents. Once DSBs are detected, a DDR is triggered to stop the cell cycle progression, thus providing time for the DNA repair machineries to act. If damages are too significant or if the repair systems are overwhelmed or inefficient, senescence or cell death will be triggered. Altogether, these reactions correspond to the DDR.

**Figure 1:**
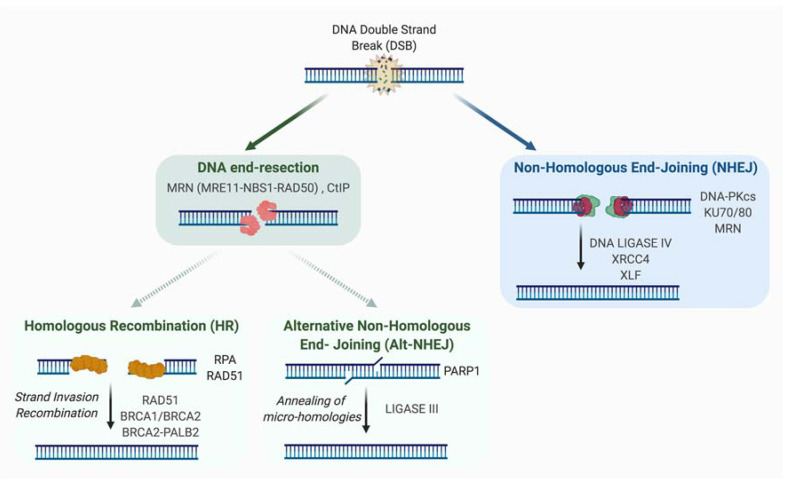
**Double-Strand Break Repair Mechanisms.** A deficient repair of DSB has detrimental consequences, including genomic instability and cell death. There are two main mechanisms to repair DSBs: HR and canonical NHEJ (C-NHEJ). A pivotal process in the choice of DNA repair pathways is the DNA end-resection. Indeed, if DNA end-resection is blocked, C-NHEJ is favored to the detriment of HR. C-NHEJ involves the ligation of DNA extremities without the requirement for homology and does not require resection. An alternative pathway of NHEJ (Alt-NHEJ) exists and unlike C-NHEJ, it is highly inaccurate and requires short DNA end-resection. Unlike Alt-NHEJ, HR requires extensive DNA end-resection and requires the presence of an intact homologous template.

Two major repair systems are involved in DSB repair: non-homologous end joining (NHEJ) and homologous recombination (HR) (Figure 1).

### 2.1. Non-Homologous End-Joining (NHEJ)

NHEJ involves the ligation of DNA extremities without the requirement for homologous sequences. Also unlike HR, it is active in all phases of the cell cycle and the canonical NHEJ pathway (C-NHEJ) does not require resection. First, DSB ends are detected by KU70-KU80 heterodimer, a ring-shaped structure associated with DNA ends, sliding inward along the DNA. A key role of KU in C-NHEJ is the recruitment and activation of DNA-PK protein, for which a role in mediating the synapsis of DNA ends was proposed [11,12]. However, other studies have shown that synapsis could be mediated independently of DNA-PK [13,14]. The catalytically active DNA-PK holoenzyme (DNA-bound KU with the catalytic subunit of DNA-PK) phosphorylates in vitro many components involved in the NHEJ process, although in vivo the phosphorylation of many factors seems to be dispensable for NHEJ. DNA-PKs also undergo auto- or trans-phosphorylation by ATM or ATR kinases. By modifying the synaptic complex and allowing accessibility of DNA ends, the phosphorylation of DNA-PKs is important for the potential processing of DNA extremities and their repair. Direct ligation of the DNA ends can be performed by the XRCC4-LIGASE IV complex, an activity that is enhanced by XLF and/or PAXX [15,16,17,18,19]. However, if necessary, enzymes such as ARTEMIS, APLF, PNKP, APRATAXIN or WERNER proteins can process DNA extremities to create ligatable ends. Of note, it has been proposed that XRCC4 and XLF by forming long protein filaments participate in the first step of NHEJ by mediating the synapsis of the two DNA extremities [13,14,20,21,22,23]. XLF and APLF bind to KU protein, and this binding has been proposed to be important for DSB repair, survival to ionizing radiation (IR) and the filament formation [24]. Mechanisms of DNA ends synapsis and processing were recently reviewed in [25].

In 2004, Guirouilh-Barbat et al. described an intrachromosomal substrate allowing the measurement of NHEJ frequency and fidelity in living cells. The use of this substrate allowed the demonstration in living cells that the mechanism of C-NHEJ itself is accurate (confirming previous data in yeast) [26]. In fact, it is the processing event, required when DNA extremities are unligatable, as in case of exposure to IR, which is mainly responsible for small losses or additions of nucleotides at repair junctions.

An alternative pathway of NHEJ (A-NHEJ) exists and unlike C-NHEJ, it is very inaccurate and leads to the production of deletions [26,27,28,29,30]. These deleterious events, described in conditions of C-NHEJ factors, deficiency could be explained by the resection of unprotected DNA ends and the subsequent hybridization of micro-homologies distal to the DNA break. Factors such as PARP1, XRCC1, and LIGASE III had been previously identified (initially by biochemical approaches) to be playing a role in this pathway [31,32,33,34]. In 2009, we and others identified the MRE11-RAD50-NBS1 (MRN) complex as a key factor in the resection step, highlighting its importance in the initiation of A-NHEJ [28,35]. Thus, we had proposed at that time that the competition between A-NHEJ and HR takes place downstream of MRN and is based on the extent of the resection [28,36]. Indeed, unlike HR, which requires extensive resection, A-NHEJ demands only short resection. Additional factors have been shown to be involved in mediating A-NHEJ and inhibiting HR such as PARP3 [37] and POLθ [38,39,40].

### 2.2. Homologous Recombination

The HR pathway is an evolutionarily conserved mechanism that participates in the preservation of genome stability and the transmission of genetic information. HR has a key role in the repair of DNA damages, such as DSBs and single stranded DNA (ssDNA) gaps. HR mechanism requires the presence of an intact homologous template, and is therefore active in S/G2 when the sister chromatid is present. Briefly, this template-dependent pathway is initiated by the recognition and binding to the broken ends by the MRN complex, which allows ATM kinase recruitment and activation. Upon ATM activation, a 2-step resection process takes place. The initial short range nucleolytic degradation mediated by the MRN/CtIP complex, favored by BRCA1, is followed by an extensive resection by other nucleases and helicases such as EXO1, DNA2, WRN, or BLM, generating long 3′ ssDNA overhangs [41,42]. These protruding ssDNA strands are preserved from degradation by binding of the RPA protein. At a later step, both BRCA2/PALB2 promote the replacement of RPA by RAD51, allowing the formation of RAD51 nucleofilament [43], which mediates homology search and strand invasion, establishing the displacement loop (D-loop). Using the homologous sequence as a template, DNA synthesis will be primed from the 3′ end of the invading strand, hence promoting D-loop migration.

**Figure 2:**
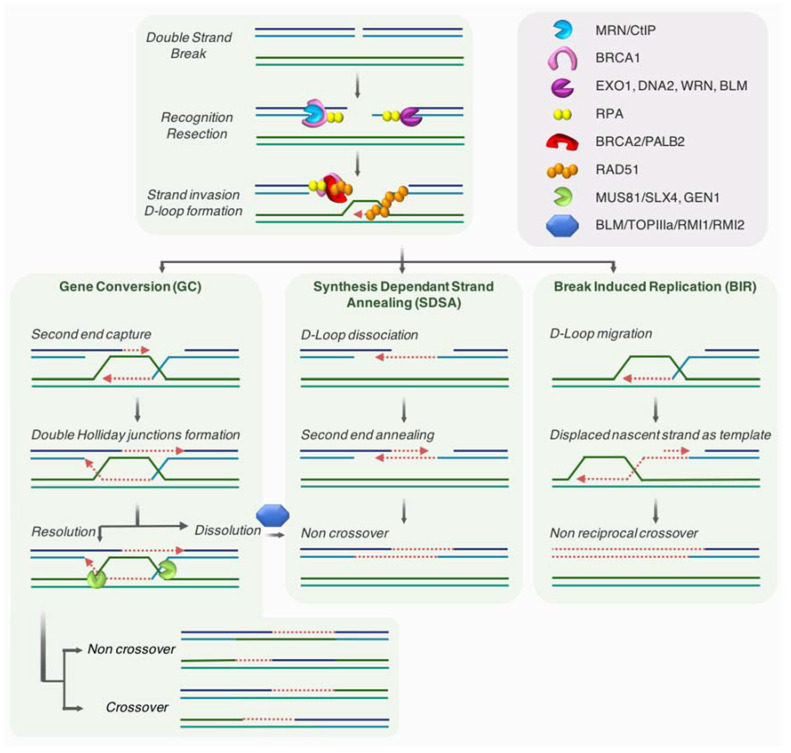
**Homologous-directed repair.** The initial resection is mediated by the MRN/CtIP complex then followed by an extensive resection by other helicases and nucleases such as EXO1, DNA2, WRN, or BLM, generating long 3′ ssDNA overhangs. These protruding ssDNA strands are preserved from degradation by the binding of RPA protein. At a later step, both BRCA2/PALB2 promote the replacement of RPA by RAD51, allowing the formation of RAD51 nucleofilament. This RAD51 nucleofilament mediates the homology search, strand invasion and D-Loop formation. Depending on how the intermediates are processed, three main mechanisms have been described as part of the HR pathway: *Left panel*: In GC, the D-loop extension promotes the annealing of the second end of the break, generating two Holiday junctions (HJ). Dissolution of this two HJ by helicases such as BLM-TOPIIIIa-RMI1-RMI2 will generate non-crossover products, while resolution through cleavage by DNA structure-specific nucleases such as MUS81, SLX4 or GEN1, can mediate both crossover and non-crossover products. *Middle panel:* In SDSA, the synthesis mediated from the invading 3′ end proceeds until it is sufficient to allow annealing to the 3′ end on the other side of the break, and synthesis of both strand goes on until their ligation with the other end of the break. The D-loop is transitory and no HJ is formed, generating non-crossover products. *Right panel:* In the BIR pathway, without a second end of the break available for the repair, the synthesis proceeds through the migration of the D-loop and covers long distance. The lagging strand uses as template, the newly synthetized strand emerging from the D-loop. This mechanism can allow the copy of the entire template, resulting in loss of heterozygosity. Blue and green DNA represent sister chromatids and the newly synthetized DNA is highlighted in red.

Depending on how the intermediates are processed, three main mechanisms have been described as part of the HR pathway [44,45,46]: gene conversion (GC, also termed double-strand break repair; DSBR), synthesis-dependent strand annealing (SDSA), and break-induced replication (BIR) (see Figure 2 for details).

Even if HR is likely a conservative mechanism, different reports proposed that this homology-directed repair is not fully accurate [47]. Indeed, BIR is largely mutagenic [48].

BIR pathway works when only one end of a break is able to find a homologous template for repair. This situation may ascend when a RF comes across nicked DNA that might convert into one-ended DSB, as well as when one of two broken DSB ends is unable to find a homologous partner. In a last instance, BIR might be employed at eroded telomeres [49,50,51]. Recent findings indicate that fragile telomeres can arise from BIR-mediated repair of telomeric DSBs [52].

Beside its crucial roles during meiosis, in concerted evolution of some sequences such as globin genes or adaptation, HR is also a vital pathway that allows cells to endure replication stress [53,54,55]. Indeed, the HR machinery not only participates in sealing ssDNA gaps during DNA replication, but also works as a fork-stabilizer and a restructurer of the replisome at collapsed forks upon stress [56]. Moreover, DNA ends or breaks occurring at stalled/collapsed RF are taken in charge by HR players to insure repair and/or resuming of the RF (see Section 3.2.2).

## 3. Replicative Stress

Many obstacles hamper the progress of replication machinery and lead to subsequent slowing or stalling of RFs. This defect in progression of normal replication is commonly called replicative stress. In this paragraph, we will present the different causes of replicative stress and how this stress is managed.

### 3.1. Different Causes of Replicative Stress

**Figure 3:**
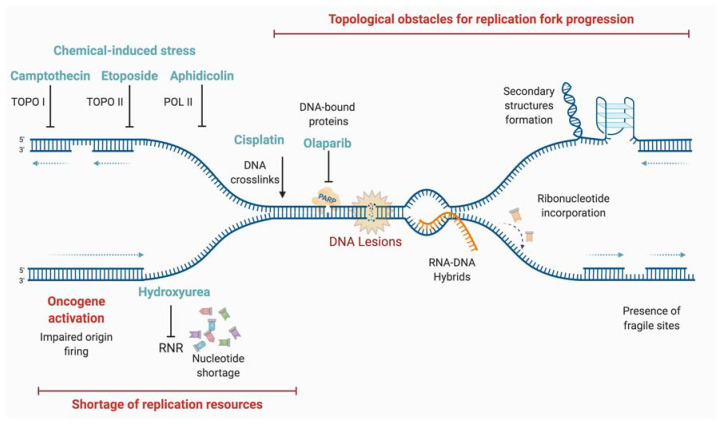
**Main sources of replication stress.** DNA replication progression is constantly challenged. Replication stress, a major source of genome instability, is defined as the temporary slowing or stalling of RFs due to the encountered obstacles or DNA lesions induced by both endogenous and exogenous agents. Among them are topological obstacles such as G-quadruplexes, the shortage of replication resources, oncogene activation, DNA protein crosslinks, and DNA-RNA hybrids (orange strand). Additionally, several chemicals can induce replication stress by the inhibition of key enzymes for replication or generation of blocking lesions (light blue).

There are different causes of replicative stress, with either endogenous or exogenous origins (Figure 3).

DNA damage, such as oxidative lesions arising from cellular metabolism, is a source of replicative stress. Other endogenous sources of replication stress are difficult-to-replicate regions such as repetitive sequences that can form DNA secondary structures, RNA-DNA hybrids formed by collisions with active transcription machineries, or DNA-protein adducts [57]. In pathological conditions, the activation of some oncogenes leads to rapid cell proliferation, exhaustion of deoxynucleotide triphosphate (dNTP) pools, and replicative stress. It has been reported that this hyper-replicative/proliferative state could be associated with a burst of reactive oxygen species (ROS) that could damage the DNA templates during replication and induce the DDR [58,59,60,61]. Finally, exogenous exposure to genotoxic agents (such as cross-linking agents), whether accidental or therapeutical, leads to DNA lesions that could impede the progression of RFs.

#### 3.1.1. Oxidative Lesions: “Natural” Causes of Replicative Stress

A large variety of barriers routinely impede RF progression. Endogenous sources of DNA damage can cause up to approximately 15,000 lesions per cell per day [62]. Among the by-products of cellular metabolism that may cause a replication stress are the ROS. These are endogenous sources of DNA damage [63] that are able to impede the progression of RFs either by directly inducing abasic sites, inter-strand-crosslinks (ICL) and oxidized bases, or by oxidizing factors of genome maintenance such as some replication components. Indeed, studies show that oxidative stress generated by H_2_O_2_ treatment can lead to the slowing [64], stalling, or reversion [65] of RFs. In addition, slowed RFs induced by the inactivation of HR proteins (without additional treatment) are rescued by antioxidant treatment showing that this impairment of RF progression is likely due to endogenous ROS [64].

To avoid genome instability, a control of replication speed occurs through the sensing of ROS at forks (such as a consequence of metabolic imbalance due to ribonucleotide reductase (RNR) fluctuation) by PRDX2 and the dissociation of TIMELESS from the replisome [66]. Consequently, the depletion of PRDX2 increases the incidence of ultrafine DNA bridges during anaphase and 53BP1 nuclear bodies during G1 phase, both of which are hallmarks of replicative stress.

Oxidized DNA bases, such as thymine glycol, can block the RF [67]. Yet, other non-blocking lesions may also cause replicative stress. Indeed, it is also suggested that the repair of oxidized bases itself could lead to RF stalling and replication stress [68]. In addition to ROS sensors such as PRDX2, glycosylases such as NEIL1 or NTH1 [69], which participate in the repair of oxidized bases by base excision repair (BER), have been also detected on RFs using the iPOND technique [70]. These glycosylases could act together with replication proteins allowing a pre-replicative repair of oxidized DNA bases. However, the repair mechanism itself (or more likely incomplete repair) may lead to DSB especially if RFs encounter intermediate repair structures (abasic sites or short patch of ssDNA). Therefore, in case of deficient or overwhelmed repair in response to an important level of oxidative damage, the level of replicative stress and subsequent DSB are increased leading to cell death. Thus, it has been recently proposed than BER proteins, such as OGG1, could be potential targets for cancer treatment [71].

#### 3.1.2. Repetitive Sequences and Particular DNA Structures

Repetitive DNA sequences, which are widely found in the human genome, challenge the replication process and act as a source of genomic instability. Among these are microsatellites, minisatellites, and satellites, which can give rise to secondary DNA structures that are different from the canonical B-DNA conformation [72]. These structures can lead to the slippage of DNA polymerases, incomplete DNA replication, or fork stalling, all of which lead to genomic instability [73]. Of note, two recent studies used genome-wide mapping to identify repetitive sequences as sites of DNA RF collapse [74,75]. Many sequences in the human genome can adopt a conformation that differs from the canonical B-DNA double helix. These particular structures, such as hairpins, cruciforms, slipped strand DNA, triplex DNA, Z-DNA, and G quadruplexes act as a barrier for RFs, leading to the stalling of replication machinery [72,76]. For instance, G-quadruplexes, four-stranded helical structures formed at GC-rich sequences, are enriched at telomeres, yet also at the proximity of numerous replication origins. These structures seem to participate in the preferential firing at those origins [77]. However, as shown upon the loss of helicases capable of unwinding these structures [78,79], G-quadruplexes represent a potential threat for the replisome and cause fork arrest [80].

#### 3.1.3. RNA-DNA Hybrids

DNA replication and transcription are fundamental processes that use the same DNA template to operate. Replication-transcription collision can lead to the formation/stabilization of R-Loops, three-stranded nucleic acid structures composed of RNA-DNA hybrids and ssDNA sequences. It is assumed that R-loops are generated from the re-hybridization of nascent RNA transcripts with their template DNA [81]. Codirectional or head-on collision are difficult to avoid, for example, at very long genes or those which are highly transcribed in S phase and are a natural source of replicative stress [82,83]. In either type of collision, the replication fork is unable to progress past the transcription machinery, but data suggest that head-on collision is more harmful than codirectional ones [84].

R-Loops are preferentially formed at GC-rich sequences and are involved in physiological processes such as the regulation of gene expression or immunoglobulin class-switch recombination [81]. R-loop are abundant and their levels and/or location are tightly regulated by mechanisms preventing their formations, stabilization, or allowing their removal. Dysfunction of these mechanisms leads to R-loop accumulation, which leads to replication stress [81]. Studies showed that RNA binding proteins, such as TDP-43, associate with nascent RNA at newly transcribed sites preventing annealing of RNA with DNA and the formation of RNA-DNA hybrids. Loss of such proteins leads to increased level of R-loops, thus promoting replicative stress [85]. 

In mammals, ATM and checkpoint kinases turn off gene transcription around DSB. However, RNA-DNA hybrids are generated at the DSB end contributing to DSB repair as long as they are transient. Nascent hybrids are involved in both C-NHEJ and HR and could act as a homologous template for DNA repair. Nonetheless, persistent hybrids have negative consequences on the repair of transcribed genes [86,87], and may lead to replicative stress. 

R-loops are associated with instability at common fragile sites [88]. They impede the replication process, as observed by the slowing and stalling of the fork, and induce DNA breaks and genomic instability [89,90,91,92].

#### 3.1.4. Oncogenes

Studies suggest that the activation of many oncogenes induce replication stress through potentially different mechanisms [93,94]. Whether by affecting origin firing, increasing replication-transcription collisions, perturbing nucleotide metabolism or inducing ROS, oncogene activation leads to replicative stress [57] and contributes to genomic instability, an important driving force of tumorigenesis [95]. For instance, shortening of the G1 phase of the cell cycle after *MYC* and *Cyclin E* oncogene activation induces an early firing of replication origins. This leads to the collapse of RFs owing to collisions with transcription complexes, therefore causing replication stress and genomic instability [96]. As described above, oxidative damages induced by ROS upon oncogene expression may also impact the progression of RF and leads to replicative stress [58,59,60,61] and could participate in genome instability. Oncogenic *RAS* expression, which results in primary human cells to oncogene induced senescence impact key DNA repair factors. Indeed, during RAS-induced senescence, an activation of CTSL and downregulation of VDR was observed, leading to a decrease of 53BP1 and BRCA1 level, which could contribute to replicative stress and genomic instability [97]. 

#### 3.1.5. Different Lesions

Unrepaired DNA lesions induced by endogenous (e.g., oxidative stress) or exogenous sources are important causes of replicative stress. Many exogenous genotoxic factors, including chemicals (DNA alkylating agents, topoisomerase inhibitors), or radiations (ionizing, UV, etc.), lead to DNA lesions [98]. Interestingly, nicks, gaps, and stretches of ssDNA are natural intermediates of many DNA processes and can be causes or consequences of replicative stress. For instance, if a RF passes through nicks or gaps, the latter would be converted to DSB, inducing fork collapse. If left unprotected, ssDNA stretches, which are not only intermediates of many repair processes but also of mechanisms promoting fork restart, can encounter endonucleases, and be converted to DSB [57].

DNA ICL and DNA-protein crosslinks (DPC) are other lesions, which also stall RFs. These ICL (and most probably also DPC) can be repaired by different mechanisms [99] including the Fanconi Anemia (FA) pathway [100]. FA is a chromosome instability syndrome, for which mutations in 22 different genes have been described, among them genes encoding for HR proteins, playing crucial roles in ICL and DSB repair, and management of replicative stress [101].

#### 3.1.6. Defective Replication Process 

Replication stress can arise from defects in the replication machinery itself, or in the components required for its function, thus hindering replication progression. A shortage in dNTP pools has been described as a major source of replication stress [102,103]. For instance, a depletion of dNTP pools can be induced by chemical treatments such as hydroxyurea (HU), or by uncontrolled replication initiation such as excessive origin firing or oncogene activation [104].

### 3.2. How Is Replicative Stress Managed?

Replicative stress is considered an important source of genome instability and may be responsible for initiation of senescence [105] or tumorigenesis [106,107]. Suitably, several mechanisms have evolved to manage replication stress, thus preventing chromosomal alterations or aberrant segregations of the genetic material. Avoiding new origin firing, protecting and promoting fork stability, repairing breaks induced during forks stalling and resuming the RF progression are the main pathway to correctly achieve a “faithful” replication process [2,55,56].

**Figure 4:**
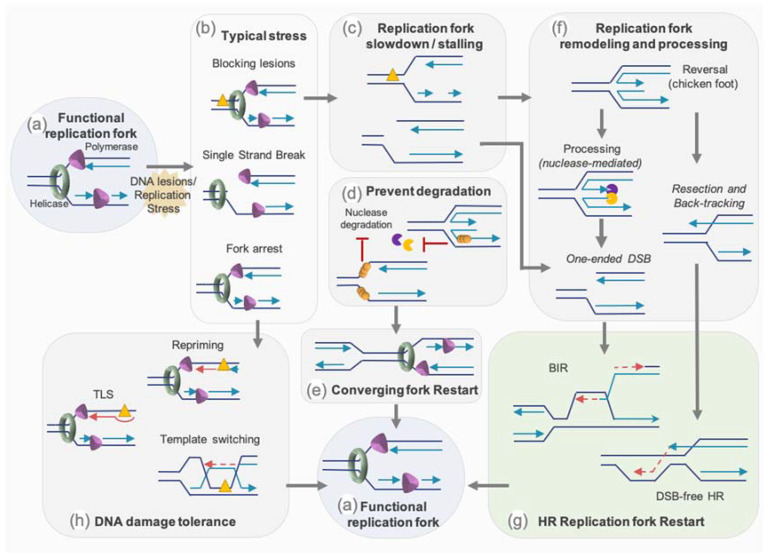
**Mechanisms of replication forks restart.** During progression, a functional RF might encounter obstacles, which will generate replication stress as a result of, for example, single strand breaks, blocking lesions, or fork arrests (**a**). This lesion will trigger the slowing of the RF or, in worst case scenario, its stalling and collapse (**b**). Protection of stalled/collapse fork from nuclease degradation (**d**), might allow replication to complete by merging with a converging fork (**e**). Persistent collapse fork will require remodeling and processing to permit the restart of the replication (**c**). One key step is fork reversal/regression, forming a 4-branch structure commonly called “chicken foot”, which will protect from extensive nuclease degradation (**d**) but also mediates both DSB-dependent or DSB-free fork restart. Controlled resection of newly synthetized DNA on a reversed fork or stalled fork, and backtracking of the fork, form a 3′-protuding end, which coated with RAD51 can mediate D-loop formation and homology search to initiate a DSB-free HR restart (**f**). One-ended DSB can arise from encounter of single strand break with the replication machinery, but also from nuclease cleavage of persistent stalled fork or reversed fork (**d**). Such one-ended DSB are then taken in charge by BIR, a specialized homology-directed repair pathway (**f**). Other types of lesions, such as blocking lesions, can trigger DNA damage tolerance pathways, which permit the bypass of sources of replication stalling and comprise three main mechanisms (**h**). Translesion DNA synthesis (TLS) involves specific DNA polymerase able to traverse a blocking lesion. Template switching is the re-annealing of the nascent strand to sequence in the newly synthetized DNA. Repriming consists of reinitializing replication downstream of the blocking lesions (**h**). Fork restart is schematized by red lines and arrows.

Protection mechanisms, especially from HR proteins, can allow the stalled fork to stand still until reached by an active progressive RF to complete the genome duplication. While obstacles on the lagging strand will mostly be “skipped” as the discontinuous synthesis of this strand will stop at and restart pass this obstacle, obstacles on the leading strand have to be dealt with. Cells have therefore settled an intricate set of mechanisms to overcome obstacles for the RF progression and to resume stalled or broken RFs. The mechanisms that are known to restart replication comprise: (1) protection and/or the remodeling of the fork to promote its restart by homology-mediated recombination mechanisms such as break-induced repair (BIR), (2) the bypass of the damage by specialized polymerases, (3) the re-priming of the replication machinery downstream of the lesion [1] (Figure 4).

#### 3.2.1. Avoiding New Origin Firing—Role of ATR

Replication stress, leading to uncoupling between the action of polymerase and helicase on the leading strand, produces long stretches of ssDNA, which is protected by RPA, forming at the same time, a signalling platform to preserve stalled fork integrity. Subsequently, it is recognized and bound by ATRIP and the ATR kinase [108,109], leading to their complex activation depending on their interacting partner as well as on DNA structure [110,111,112,113,114,115,116,117,118]. ATR replication checkpoint activation allows the recruitment of DNA repair proteins, prevents dormant origin firing, promotes RF stability, and process for replication restart [119]. Inappropriate firing would lower the pool of replisome components and might deprive active origin from those. Moreover, inefficient ATR activation or origin firing deregulation leads to an increase of ssDNA regions and therefore to the exhaustion of RPA. The unprotected ssDNA regions at the fork induced its collapse and are more prone to nucleolytic cleavage, leading to potential DSBs or forks cleavage [120]. The ATR replication checkpoint participates in RF stabilization and prevents their collapse. Fork collapsing leads to the formation of DSB and the inability to resume replication with the consequence of potential incomplete DNA replication [121,122].

#### 3.2.2. Restart by Homology-Mediated Recombination

The HR mechanism and factors play essential roles in reestablishing functional RF (Figure 4). Besides their role in the homology-directed recombination process, some HR proteins protect stalled fork and nascent strand from degradation by nucleases (see Section 3.2.3). These factors also protect and participate in the remodeling of stalled fork, such as the conversion of the 3-branch structure into a 4-branch structure by reannealing of the two parental strands and backtracking and annealing of the two newly synthetized strands. This molecule resembling a Holliday junction and called reversed or regressed forks, have been visualized by EM and are increased after different genotoxic stress such as replication stress-inducing agents: DNA synthesis inhibitors, topoisomerase inhibitors, DNA interstrand cross-linking agents, alkylating agents, UV radiation, and oxidative stress [65].

Fork reversal, which is seen as a mechanism of protection from degradation (see Section 3.2.3), might also be a way to allow the repair of the lesion on a non-fork structure, but also to reinitiate a functional D-loop without inducing DSB. As a matter of fact, the mechanistic of RF restart through homology-mediated recombination can happen independently of the induction of DSB or following such an event [54,55]. Indeed, regression and controlled resection of a reversed fork, or resection of the nascent strand of the lagging strand on a stalled fork, and backtracking of the fork might generate a 3′ protruding overhang. Evidence in yeast suggest that, as for classical HR, DSB-free fork restart through homology-mediated recombination step is mediated by short range resection by the MRN/Ctp1 (CtIP ortholog) complex followed by extensive resection through the action of Exo1 [123].

However, a RF coming across a nick will convert this lesion into a one-ended DSB. Moreover, persistent stalled RF or reversed fork might also be cleaved by nucleases or structure specific endonucleases (SSE) such as MUS81, which will generate a one-ended DSB (see Section 3.2.4). In these situations, replication is resumed by Break-Induced Replication (BIR). Although in eukaryotes, this process has been mostly described in yeast, growing evidence of the involvement of such a mechanism are reported in mammals [49,124]. In yeast, resection of the one-end DSB generates a 3′ ssDNA, which will first be coated by RPA before being converted by Rad52 into a Rad51 nucleofilament. This will trigger the homology search followed by strand invasion to initiate the formation of a D-loop. The DNA synthesis then resumes and progresses through the migration of the D-loop, with the newly synthetized strand emerging from the D-loop serving as a template for the lagging strand. BIR replication is therefore asynchronous and conservative, in contrary to classical synchronous and semi-conservative replication. In the absence of Rad51, the formation of the D-loop relies on the single strand annealing activity of Rad52. Mammalian BIR is less characterized mechanistically but it involves POLD3 and RAD52 and seems less-dependent on RAD51 than yeast BIR [49,124].

#### 3.2.3. Protection of Stalled or Reversed Forks

To prevent harmful scenarios such as the degradation of the fork, inappropriate repair or the presence of under-replicated stretches of DNA, RFs must be protected during the time required for managing their accurate restart and/or removal of stress.

The HR factors BRCA1/2 and RAD51 have a primordial role for fork protection and maintaining its stability [56]. Indeed, stalled fork degradation, as observed by the nascent strand nucleolytic processing, was reported in setting with deficiency in these HR proteins. BRCA2/PALB2, with the participation of BRCA1/BARD1, load RAD51 on ssDNA at stalled forks. Evidence from *BRCA2* deficiency and BRCA2 S3291A mutations, interfering with RAD51 interaction, indicates that the nascent ssDNA degradation by MRE11 could be due to a destabilization of the filament of RAD51 on this nascent ssDNA [125]. Additionally, it was proposed that in the absence of BRCA2, resection might be initiated by CtIP and MRE11 and a subsequent extensive processing by EXO1 [126]. Recent studies also direct towards a role for BRCA1/BARD1 in the protection of stalled fork from MRE11 by improving filament stabilization by RAD51 [127,128]. Depletion of RAD51 or preventing RAD51 binding to DNA causes degradation of nascent ssDNA by MRE11 and affects the RF restart [125,129,130,131]. However, the strand exchange activity of RAD51, which is primordial for HR, is not required to protect the fork from MRE11 degradation [132].

Fork reversal is considered as a protective mechanism against genomic instability, avoiding excess of ssDNA. However, as for non-regressed forks, mechanisms of protection at reversed forks should be active, since this structure composed of an one-ended extremity is prone to nuclease processing (e.g., by MRE11/CtIP, EXO1…) and DSB formation (e.g., by MUS81 nuclease) (Figure 4).

Different proteins are proposed to participate in fork reversion [56,133]. Reversed forks are decreased in BRCA1/2 deficient backgrounds. However, upon MRE11 depletion, fork reversion can be restored [126,131]. Likewise, EXO1 depletion in BRCA2 deficient cell lines is able to rescue fork reversal [126]. The RAD51 depletion mimics BRCA2 depletion in decreasing reversed forks number, with the exemption that, in RAD51 depletion case, MRE11 inhibition has no effect in rescuing this number, confirming the crucial role of RAD51 in fork reversal [65,131,134]. Although not essential for fork reversal, a stable RAD51 filament is required for the reversed fork protection from degradation [131]. Interestingly, contrary to the role of CtIP in initiating nascent strand processing at stalled fork, the nuclease activity of CtIP seems to counteract at reversed fork the excessive degradation mediated by DNA2 [135]. Studies reported that fork remodelers, such as ZRAN3B and SMARCAL1, promote the MRE11 dependent degradation of reversed fork structure in the absence fork protector such as BRCA1/2, RAD51 or FANCD2 [136]. It has been proposed that RAD51-BRCA2 and others proteins such as BOD1L, FANC could form a complex, a “protectosome”, which would protect the RFs [137].

NHEJ was also proposed to play a role in managing DSB during S phase or replicative stress [138,139,140]. Recent evidence also implicates NHEJ factors in the protection of the RF. Indeed, in yeast, the NHEJ component KU, acts as a protector of the reversed RF [123]. In mammalian cells, the NHEJ factor RIF1 protects reversed fork from DNA2 degradation [141]. Moreover, XLF and H2AX seems to play a successive role in preventing reversed fork formation and in protecting from MRE11 degradation, the nascent strand of regressed arms [142].

Once the forks are protected and stabilized, the action of nucleases and/or helicases is necessary for resuming the replication.

#### 3.2.4. Nucleases and Helicases Mediating HR Restart of the Fork

Nucleases play key roles in DNA repair mechanism but also in the processing of stalled RF and their efficient restart. While limited resection and/or DNA cleavage are required for efficient fork restart, extensive degradation of stalled replication intermediate has to be prevented, as it is a source of genomic instability. Upon replicative stress, MRE11 could also play a role in fork restart and prevent DSB formation [143]. Indeed, it has been suggested that MRE11 allows the enlargement of gaps of ssDNA, which increase the amount of ssDNA template for RAD51 loading and efficient repair [144]. At the same time loading of RAD51 on ssDNA would prevent uncontrolled resection by MRE11, which leads to the formation of large ssDNA gaps and genetic instability [129]. Aside for MRE11, FAN1 [145] and CtIP [146] have also been implicated in the restart of the replication, when under appropriate control by FANCD2, as in its absence, excessive processing by FAN1 is observed [145]. The gap-endonuclease activity of FEN1 also seems to mediate restart of stalled RF [147].

If the fork stalling is prolonged and/or failed to restart, it might become a substrate for structure specific endonuclease (SSE). Theses SSE process DNA secondary structures that can be produced during DNA repair or DNA replication, such as stalled RF or reversed fork [148]. Among them, the SLX4-MUS81-SLX1 complex participates in RF cleavage and in the generation of single-ended DSB. Indeed, SLX4 has been associated with DSB formation at prolonged stalled fork under certain circumstances [149,150,151], but many studies point more toward MUS81 as a key player in the restart of stalled RFs mediated by their cleavage into DSB [152,153,154,155,156]. Whether this function of MUS81 relies on its SLX4-dependent recruitment is not clear and might depend on the type of replication stress induced [151,157]. Nonetheless, after extensive replication stress, SLX4 itself is sufficient to avoid GEN1 mediated fork cleavage and prevent genome instability [151]. Of note, SLX4- or MUS81- DSBs induction have been reported to have a detrimental effect for cell survival upon HU treatment [149,158]. It has also been proposed that the cleavage by MUS81 of reversed fork with limited MRE11-resection allows replication restart and survival in BRCA2-deficient cells [126]. Other data suggest that following ATR inhibition, excessive SMARCAL1 activity results in increased fork reversion, whose cleavage in a SLX4-dependent manner would affect cell viability and fork progression [157].

While helicases from the RECQ family can resolve secondary structure such as stalled RF, they can co-operate with nucleases to promote the RF restart. Indeed studies suggest that WRN might acts with FEN1 at stalled RF [147,159,160]. In addition, it has been reported that BLM contributes with MUS81 to convert stalled RF into DSB, to promote the repair and resumption of the RF [153,161]. Under conditions of prolonged RF stalling, DNA2 and WRN are important for RF restart. Indeed, DNA2 and WRN are essential to mediate reversed fork restart by limited degradation, kept in control by RECQ1 [162]. RAD51 depletion antagonizes this mechanism [162], probably due to the fact that RAD51 plays an important role in the fork reversion [65].

Nonetheless, if these SSE and helicases are a source of DSB and genetic stability during replicative stress, they are more certainly tightly controlled, as suggested by the chromosome shattering and cell death observed following uncontrolled MUS81-SLX4 interaction and action at stalled RF [104,163].

#### 3.2.5. Translesion Synthesis and Repriming

Depending on the type of lesions encountered during replication, DNA damage tolerance pathways can permit their bypass through the three main mechanisms: translesion DNA synthesis (TLS), template switching or repriming [164]. DNA damage tolerance can be achieved by either specialized DNA polymerases or by delaying repair. To guarantee the replication of the damaged template, TLS involves the action of lesion-bypass DNA polymerases (POLη, REV1, POLκ, POLι, POLν, REV3L-REV7, and POLθ) to bypass the fork-associated lesions [165,166]. These polymerases have unique features that allow them to synthesize DNA past damaged bases [165].

Template switching corresponds to a realignment of the nascent strand to somewhere else in the newly synthetized DNA sequence to use as a template, such as the sister newly synthetized strand. Template switching can also occur in fork reversal to replace the obstacle into a conformation that will allow it to be “bypassed” as synthesis uses regressed arm as a template. The classical fork progression is then restored either by strand processing of reversed arm and annealing to sister nascent strand in an HR-dependent fork synthesis restart or, by restoring the initial fork nascent and parental strand position [167].

Alternatively, the replication machinery can skip damaged DNA and reprime the fork downstream of the lesion. The primase/polymerase PRIMPOL is able to synthesize primers and promote the DNA synthesis restart when fork progression is challenged [168,169]. PRIMPOL repriming generates post-replicative ssDNA gaps. The efficient filling of post-replicative gaps requires the action of either TLS polymerases or strand exchange and template-switching mechanisms. Under stress conditions, the activation of PRIMPOL can counteract pathological reversed fork degradation, by reinitiating DNA synthesis past DNA lesions [164]. Recently, the PRIMPOL pathway has been proposed as a therapeutical target in BRCA1-deficient cancer [170]. This study shows that PRIMPOL is involved in an adaptive response to genotoxic stress. Indeed, upon cisplatin pretreatment, ATR induces an increase of PRIMPOL expression, which leads to decreased RF reversal, and an increase in PRIMPOL-dependent repriming, thus promoting cell survival. This balance between fork reversal and repriming allows cells to cope with cisplatin-induced DNA lesions [170]. Targeting both of these pathways could increase cancer cell chemo-sensitivity.

### 3.3. Responses to Avoid Genome Instability upon Replicative Stress

Absence or insufficient management of replicative stress leads to genome instability and could even result in a chronic inflammatory cellular environment, which is associated with different human diseases, such as neuropathologies, age-related disorders, and cancers.

#### 3.3.1. Nature of DSB during Replicative Stress and the Role of Sister Chromatid Cohesion

The particular nature of DSBs during replication stress is highly prone to result in chromosomal rearrangements. Indeed, in contrast of the “two-ended” DSBs, “one-ended” DSBs do not always have the possibility to be accurately joined with a proximal DSB and can potentially be joined to other distal DSBs, leading to translocations or complex chromosomal rearrangements, such as chromosome fusions or radial figures. Radial figures are observed in cells deficient for HR, in BRCA1 or BRCA2 mutated tumoral cells for instance [171,172,173,174] or in Fanconi pathway-deficient cells [101]. A correct sister chromatid cohesion is capital to avoid genome instability. Cohesins play here a crucial role to avoid mobility of the “one-ended” DSB extremities. Gelot et al. showed that RAD21 depletion leads to an increase of distal end-joining and an increase of chromosome rearrangements such as translocations [175,176]. In addition, the human TIMELESS-TIPIN complex, which is involved in RF stabilization/protection, S-phase checkpoint activation and establishment of sister chromatid cohesion [177], helps to prevent genome instability [178].

#### 3.3.2. Mechanisms to Avoid Replication Stress-Induced Chromosome Aberrations by Precluding NHEJ Factor or by Positively Regulating HR

Different sophisticated mechanisms exist to preclude end-joining mechanism in case of unresolved replicative stress to avoid chromosome aberrations or telomeres fusions. For instance, the recruitment of 53BP1, which favors NHEJ by inhibiting end resection with RIF1 and shieldin proteins [179], is impeded at mitosis by PLK1 kinase. The phosphorylation of 53BP1 UDR region inhibits its binding to H2AUb in chromatin. In contrast, its dephosphorylation by PP4C protein in G1 allows this protein to be recruited to damaged chromatin [180,181,182]. The absence or deficiency of BRCA1 (or other HR proteins as FANC proteins), leading 53BP1 to be active during replicative stress, leads to complex chromosome aberrations, as radial figures [139,140,171,183,184]. However, a PLK1-dependent temporally competition between 53BP1 and BRCA1 to restart stalled fork exists. Indeed, while depleting BRCA1 mediates, a 53BP1-dependant DSB-free fork restart, depletion of 53BP1 leads to a restart by BIR following a SLX4-MUS81 cleavage [185]. This suggests a tight control of 53BP1 recruitment during replication stress/or in each cell cycle phase. Thus, the choice between different DSB repair mechanisms; HR vs NHEJ, is capital to ensure genome stability. Resection, which is very tightly controlled, favors HR at the expense of NHEJ during the S phase. For instance, regulation of key proteins in DSBR, such as CtIP or BLM favors resection or HR during the S phase. We reported that BLM has a dual role: during G1, it avoids large resections by its binding to 53BP1; in contrast during the S phase, BLM binds to TOPIII, which results in its increase of unwinding activity and subsequent resolution of DSB (or replicative stress) by HR [29]. Another example, as cells enter the S phase, phosphorylation of Sae2/CtIP by CDK1 occurs, allowing its binding to BRCA1 and MRN complex leading to a stimulation of resection, not only through MRN activation but also by displacing 53BP1, the factor antagonizing resection [186,187].

#### 3.3.3. Consequences during Mitosis

More than one hundred years ago, Theodor Boveri predicted in his publication “Concerning the Origin of Malignant Tumours“ that chromosomal instability, resulting from an improper “splitting” of chromosomes, is a key hallmark of cancer [188]. Now, we know that unresolved replicative stress is a major source of chromosome segregation defect at mitosis. Slowed fork progression in HR-deficient cells (spontaneously without additional treatment) has been shown to elicit an alteration of progression in metaphase and in an increased centrosome number, leading to multipolar mitosis [189,190,191,192]. Recently, it has also been reported that replicative stress leads to premature centriole disengagement causing multipolar spindles, lagging chromosome, and micronuclei [193].

However, at difficult-to-replicate regions, such as common fragile sites or telomeres, replicative stress can have detrimental consequences such as under-replicated regions. Studies have identified a mitosis DNA synthesis termed MiDAS initiated at such regions to permit their complete replication [194,195,196,197,198]. This RAD51-independent, RAD52 and POLD3 dependent mechanism appears to result from a MUS81-SLX4 cleavage of a stalled RF as the cells proceeds to mitosis.

A review in this special issue by Wilhelm et al. described the major consequences of replicative stress during mitosis and its role in chromosomal instability (CIN) [199].

### 3.4. Consequence of Replicative Stress on Inflammation

**Figure 5:**
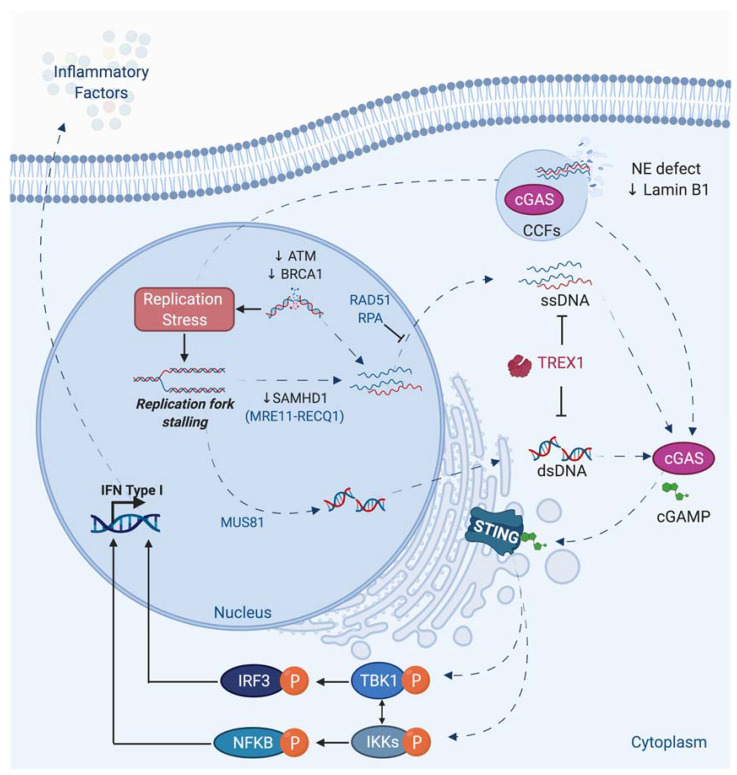
**Activation of canonical STING Pathway upon replication stress or DNA damage**. Self-cytosolic DNAs arise from genetic instability caused by endogenous (ROS, replication stress, retroelements, etc.), exogenous sources (therapeutics agents, IR, etc.) or DNA damage persistence (loss of ATM, BRCA1, etc.). Cytosolic DNA could be DNA repair mechanism by-products or generated during stalled RF processing. Indeed, MUS81 participates to RF processing and leads to double strand cytosolic DNA accumulation. It has also been reported that aberrant fork processing occurs in the absence of SAMHD1, which leads to the accumulation of cytosolic ssDNA. This is the result of the displacement of the nascent DNA strand by RECQ1 and its cleavage by MRE11. To avoid cytosolic DNA accumulation, DNA repair protein such as RAD51 and RPA are bound to ssDNA to prevent their cleavage and passage in the cytoplasm. Once in the cytoplasm, self-DNA (or cytosolic DNA originated from pathogens sources), if not degraded by TREX1, is sensed by cytoplasmic sensors such as cGAS. Cytosolic DNA are also generated from ruptures of the NE or leakage from micronuclei. cGAS activation leads to the production of cyclic GMP–AMP (cGAMP), a second messenger capable of activating the adaptor molecule STING that resides in the ER. Upon activation, STING translocates to the Golgi where it can recruit kinases IKK and TBK1. Subsequently, these proteins activate NF-kB and IRF3, respectively. These later translocate to the nucleus leading to the transcription of inflammatory factors to trigger the immune response in a type I IFN-dependent manner.

Beside its direct consequence in chromosomal rearrangements induction, replicative stress has been linked to inflammation. Indeed, during replicative stress, small fragments of DNA could potentially be produced and released into the cytoplasm (Figure 5).

The exact mechanism of genomic DNA fragments production and release is still not fully understood. The presence of these DNA fragments in the cytosol can activate a part of the innate immune system through the expression of interferon and inflammatory factors. Among different nucleic acids sensor, the DNA sensor cGAS recognizes cytosolic DNAs and produces the cyclic dinucleotide cGAMP, which is responsible for the activation of STING protein. The subsequent phosphorylation of TBK1 and NF- activation leads to type 1 interferon and cytokines production [4,5,200,201,202] (Figure 5). The activation of this innate immune system could be considered as a DDR response, since this pathway in response to damage is generally beneficial by recruiting immune cells to eliminate cells bearing damaged DNA or genome instability. However, a chronic activation of this cGAS-STING pathway and the subsequent inflammation are at the origin of inflammatory pathologies such as Aicardie-Goutières [5] and could also participate in aging or tumorigenesis [203,204,205]. Its activation could favor proliferation or propagation of tumor cells, likely by creating an inflammatory tumoral micro-environment.

Different genotoxic stress leads to cGAS-STING activation, but often the precise molecular mechanism is not fully elucidated [206,207]. 

Interestingly, a link between BRCA1, BRCA2, MUS81, or SAMHD1, different proteins playing an important role during replicative stress, and cGAS-STING activation has been reported (Figure 5).

It has been shown that deficiency in BRCA1 or BRCA2 leads to activation of the STING pathway [208,209,210]. MUS81 participates in the cleavage of genomic DNA leading to cytosolic-dependent activation of STING and interferon production. This activation promotes phagocytic and T cell responses and rejection of prostate tumors via mechanisms partly depend on macrophages [211]. SAMHD1, a protein mutated in the inflammatory disease, Aicardi-Goutières syndrome, acts at stalled RFs to prevent interferon induction. Indeed, SAMHD1 is important to control correct resection of nascent DNA, allowing the forks to restart replication. In contrast, its deficiency leads to the RECQ1-dependent release of DNA fragment from stalled forks to cytosol and to an inflammatory response [212]. An extensive review in the same issue describes the different links existing between DNA repair defect, replicative stress, and inflammation [4].

Interestingly, the cGAS-STING-TBK1 pathway, through recognition of DNA in micronuclei, was reported to participate to senescence and the Senescence Associated Secretory Phenotype (SASP) [6,213,214,215,216] and thus likely to aging [203]. It is now accepted that “inflammaging”, induced by accumulation of senescent cells likely due to less-efficient DNA repair and immune systems, is responsible for many pathologies associated with old age such as atherosclerosis, arthritis, cancer, etc. [217].

In the next section, we discuss how structural proteins such as lamins, essential components of the nuclear envelope (NE), and the nuclear matrix, have roles in DNA repair and during replicative stress. Expression of a pathological form of Lamin A protein, Progerin, which is at the origin of a very severe premature aging syndrome, Hutchinson-Gilford progeria, also leads to replication defects and an inflammatory phenotype through the cGAS-STING pathway.

## 4. Presentation of Lamins

The nuclear lamina (NL), which represents a major component of the NE, is composed mainly of the lamins, along with integral proteins of the inner nuclear membrane. Based on sequence homologies and biochemical properties, lamins are classified into two types: A and B. These type V intermediate filaments, which form a fibrous network that line the inner part of the NE, are also present in a relatively smaller yet significant fraction throughout the nucleoplasm [218,219,220]. Lamins residing in both compartments, the NL and the nucleoplasm, play important roles in various nuclear functions.

### 4.1. Two Types of Mammalian Lamins

Mammals have three lamin genes that encode for four major and three minor lamin isoforms. The major A-type lamin isoforms, A and C, and the minor A-type lamin isoforms, AΔ10 and C2, arise from a single gene, *LMNA*, through alternative splicing. B-type lamins arise from two genes, *LMNB1* and *LMNB2*. The *LMNB1* gene encodes the major isoform B1 while the *LMNB2* gene encodes the major isoform B2 and the minor isoform B3 [221,222]. Lamins A and C are expressed mainly after the tissue differentiation stage of development, while Lamin AΔ0 is expressed in somatic cells, Lamin C2 seems specific to germ-lines. In mammals, Lamin B1 and Lamin B2 are ubiquitously expressed along differentiation in germ-line and somatic cells. Lamin B3 is specifically expressed in germinal cells.

### 4.2. Maturation of Lamins

**Figure 6:**
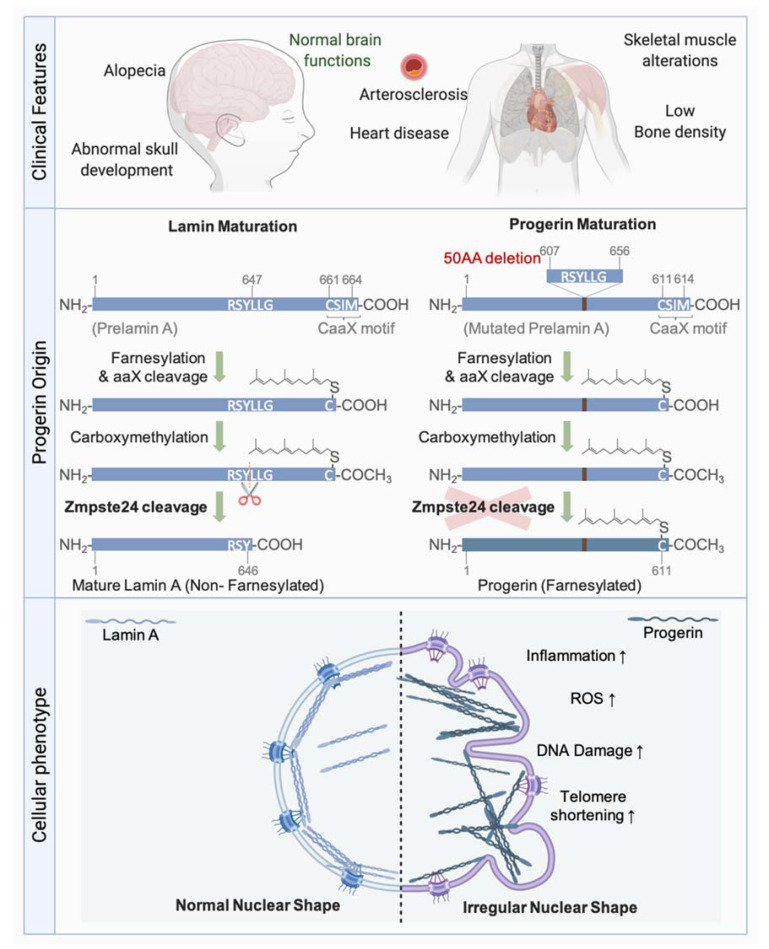
**Hutchinson Gilford Progeria Syndrome.** Main clinical features of progeria syndrome (Upper panel). Prelamin A protein goes under several post-translational modifications on the CaaX motif: addition of a farnesyl group on the cysteine residue, cleavage of the aaX, and carboxymethylation of the cysteine residue. The final step is the cleavage by ZMPSTE24 endonuclease to produce a mature Lamin A non-farnesylated nor carboxymethylated. HGPS mutation of *LMNA* results in the deletion of 50 amino acids including the cleavage site for the endonuclease ZMPSTE24 (middle panel). Thus, the resulting protein, Progerin, is permanently farnesylated and carboxymethylated. Its accumulation on the NE is associated with nuclear shape alterations. Additional cellular phenotypes of Progerin accumulation comprise increase of DNA damage, ROS, inflammation, and telomere shortening (bottom panel).

Lamin precursors undergo several modifications after their synthesis, thus, allowing their maturation (Figure 6). Soon after their synthesis, all lamins except Lamin C, which lacks the final two exons found in Lamin A including the CaaX motif, undergo a farnesylation of the cysteine residue of the CaaX motif by farnesyltransferase. Following the addition of farnesyl, a C15 lipid, the aaX residues are removed by specific proteases. This cleavage is catalysed by FACE2 (also known as RCE1) in the case of the B-type lamins, and by ZMPSTE24 (also known as FACE1), a zinc metallo-endoprotease, in the case of Lamin A. Carboxy-methylation of the Cys residue at the CaaX motif by the carboxylmethyltransferase ICMT then follows. In contrast to B-type lamins, which remain permanently farnesylated and carboxy-methylated, Lamin A undergoes an additional ZMPSTE24-mediated cleavage step, which removes 15 amino acids. This final step removes the farnesylated and carboxy-methylated Cys residue, leaving a Tyr residue at the carboxyl end of Lamin A, rendering the latter mature [223] (Figure 6).

Numerous mutations, dysregulation in expression, and failure in maturation of lamins lead to many severe disorders, termed “laminopathies”.

### 4.3. Associated Pathologies

*LMNA* is one of the most frequently mutated, disease-associated genes known to date. More than 500 currently reported mutations in the *LMNA* gene (http://www.umd.be/LMNA/ accessed on 31 January 2020) give rise to at least 12 disorders. These diseases can be classified into four major categories: striated muscle diseases (muscular dystrophy and cardiomyopathies), lipodystrophy syndromes, peripheral nerve disorders, and premature aging syndromes [222,224,225,226,227].

The most emblematic premature aging disease Hutchinson-Gilford Progeria syndrome (HGPS) is caused by a *LMNA* mutation preventing the conversion of farnesyl-prelamin A to mature Lamin A [228,229] (Figure 6). The most common mutation leading to HGPS is a de novo, dominant, and silent single-base substitution in exon 11 of the *LMNA* gene leading to the activation of a cryptic splice site and a subsequent elimination of 50 amino acids. The missing residues included the second internal cleavage site of ZMPSTE24. This gives rise to an immature form of Lamin A termed Progerin, which remains farnesylated and carboxy-methylated, and accumulates at the nuclear periphery. 

Accumulation of farnesylated forms of Lamin A due to ZMPSTE24 deficiency is also toxic and leads to severe progeroid syndromes. Interestingly, PrelaminA and Progerin accumulate in normal aging cells [230,231,232,233,234], suggesting that these proteins contribute to normal aging.

Diseases associated with B-type lamins are very rare. Yet, a duplication of the *LMNB1* gene leads to autosomal-dominant leukodystrophy (ADLD) [235,236]. ADLD is a fatal demyelinating neuropathy of the central nervous system that is sometimes associated with ataxia, in addition to cardiovascular and skin defects. Increased expression of Lamin B1 in oligodendrocytes leads to reduced expression of lipid synthesis genes, resulting in reduced synthesis of myelin [237,238]. However, the tissue specificity of phenotypic expression of *LMNB1* duplication is not understood. 

An increase in Lamin B1 levels has also been reported in Ataxia telangiectasia (AT) and Werner syndrome (WRN) cells [239,240]. In addition, as we will see below, an increase in Lamin B1 is also observed in many tumors. Recently, dominant pathogenic variants in LMNB1 and LMNB2 have been identified as genetic causes of primary microcephaly, involving the NL in its etiology, and defining a new form of laminopathy [241]. Besides, de novo variants were recently identified in the *LMNB1* gene leading to disorganization of the Lamin B1 NL and to nuclei shape alteration in patients with microcephaly [242].

Several mutations in *LMNB2* are associated with acquired partial lipodystophy (also called “Barraquer-Simons syndrome”) involving the loss of adipose tissue [243]. Another homozygous missense mutation in *LMNB2* was identified in certain patients with autosomal recessive progressive myoclonus epilepsy with early ataxia syndrome, perhaps due to abnormal neuronal migration [244].

Furthermore, a study showed that loss of B-type lamins can directly lead to age-related neurodegeneration, as seen in Alzheimer’s disease and related tautopathies, suggesting that these diseases are in fact acquired neurodegenerative laminopathies. In neurons of human brains affected by Alzheimer’s disease, pathological tau causes the stabilization of actin filaments, thus leading to the disruption of the LINC complex and the reduction of B-type, but not A-type lamins. In return, this leads to the relaxation of constitutive heterochromatin and activates the cell cycle in post-mitotic neurons, which leads to their death [245].

### 4.4. Lamins and Senescence

As seen in several progeroid syndromes and during the aging process of wild-type cells, nuclear shape alterations (NSA) occur commonly during senescence. The premature senescence, observed in cells from several progeroid syndromes including HGPS and also AT and Werner syndrome (with different degree of aging phenotype), depends, at least partially, on a dysregulation in lamins. It has been suggested that an unbalanced ratio between A- and B-type lamins might direct cells into senescence [246]. Interestingly, studies also reported changes in lamin levels during senescence and during the normal aging process.

#### 4.4.1. A-Type Lamins and Senescence

Many studies have proved that the defective maturation of Lamin A or alterations in its levels result in premature entry into senescence. For instance, the silencing of Lamin A with shRNA [247] or its overexpression [248,249] leads to cell cycle arrest and senescence induction. Interestingly, the overexpression of the Prelamin A processing enzyme ZMPSTE24, or treatment with farnesyltransferase inhibitors, improves the growth capacities of cells overexpressing wild type Lamin A. This indicates that the accumulation of farnesylated Prelamin A intermediates contributes to the senescent phenotype of these cells [249]. Indeed, other studies demonstrated that the accumulation of Prelamin A, or progeria-causing lamin A mutants such as Progerin, leads to NSA and senescence induction [248,250,251]. Notably, treatment of Progerin-accumulating HGPS cells with farnesyltransferase inhibitors also restores many of the observed progeroid phenotypes including NSA and senescence, except for the persistence of DNA damages. In addition, analysis of tissues isolated from HGPS and ZMPSTE24-deficient mice revealed an upregulation of SASP genes, which were suggested to contribute to the systemic inflammation observed in these mice [252]. Since an accumulation of Prelamin A or Progerin has been observed in normal aging cells [230,231,232], it is suggested that these proteins are implicated in the normal aging process. Progerin and PrelaminA expression have a detrimential effect on vascular cells due to diverse molecular causes. HGPS was proposed as a model of vascular aging [253]. Progerin expression leads to an increase of oxidative stress, inflammation, DNA damage persistence, and senescence, leading to endothelial cells dysfunction [254]. This could contribute to HGPS-associated early atherosclerosis. Accumulation of Prelamin A in smooth muscle cells promotes the calcification and aging of these cells and induces the activation of the SASP [255]. 

#### 4.4.2. B-Type Lamins and Senescence

Cells depleted from Lamin B1 exhibit nuclei with deformed shape and enter into senescence [256,257,258,259,260,261]. Several studies reported a decrease in Lamin B1 expression during multiple types of cellular senescence, including normal aged tissues, and replication-, irradiation- or oncogene-induced senescence [258,260,261,262,263,264]. Early at the onset of irradiation-induced senescence, the downregulation of Lamin B1 along with the lamin B receptor are necessary for the release of heterochromatin binding to the NL [264]. Lamin B1 depletion during senescence is coupled to global and local changes in chromatin methylation [260,261], such as formation of domains depleted for the repressive histone mark H3K27me3, and is correlated with the upregulation of key senescence genes, including the SASP genes [261]. However, we and others showed that an increase in Lamin B1 also leads to an alteration in nuclear shape and senescence induction [239,263,265]. We also demonstrated that the increase in Lamin B1, in response to oxidative stress, contributes to the senescence phenotype of AT cells [239].

### 4.5. Lamins and Cancer

The deformation of nuclear morphology has been identified as a characteristic of cancer cells since the 1800s by Sir Lionel Beale. Although these alterations served for decades as cancer diagnostic tools, and even as prognostic markers [266,267,268], very few reports have provided insight into how these nuclear abnormalities would be induced, and what their biological consequences would be in the context of malignancies. Different studies reported deregulations of Lamin A expression in different types of cancers. A downregulation of Lamin A is more often observed in tumors than an upregulation. As example, loss of Lamin A correlates with loss of differentiation and higher proliferation in ovarian cancers. Lamin A was suggested to be a potent cancer biomarker that can be used to check tumor progression and prognosis [269,270]. Concerning B-type lamins, the dominating phenotype that is observed is an increased expression, often associated with bad prognosis. For instance, increased Lamin B1 level in pancreatic cancer has been correlated with poor prognosis, and Lamin B1 has been proposed as a therapeutic target. Indeed, the knockdown of Lamin B1 significantly attenuated the proliferation, invasion, and tumorigenicity of pancreatic cancer cells [271]. Patients affected by colon cancers or clear-cell renal cell carcinoma or gastric cancer with high Lamin B1 expression also exhibit a poor prognosis [272,273,274]. A recent report proposes that high level of Lamin B2 could promote migration of non-small cell lung cancer by increasing H3K9me2 level, which induced *E-Cadherin* gene silencing [275]. High level of Lamin B2 was also observed in triple negative breast cancer (TNBC) tissues. The depletion of LMNB2 suppressed proliferation and induced apoptosis and inhibited tumor growth of TNBC cells in mice. The authors suggest that Lamin B2 may promote TNBC progression and could serve as a potential therapeutical target [276].

The impact of Progerin expression, decreased or increased Lamin A levels, or depletion of Lamin B1 on genome stability and on inflammation, another important hallmark of cancer [277], has started to emerge.

## 5. Lamins and Genome Stability: Their Roles during DNA Repair, Replication, or Replicative Stress

Beside their important role of ensuring a good shape of the NE, and its reformation at the end of mitosis, many other functions of lamins have been described: genome organization; differentiation; cellular migration; cell cycle regulation; autophagy and apoptosis; and oxydative stress management. Different roles for lamins in DNA metabolism were also described, such as regulation of gene expression (by tethering chromatin to the NE, a repressive compartment for transcription, or by binding specific transcription factors). These different functions will not be described here as they have been extensively reviewed [218,219,220,278,279]. We will focus on the role of lamins in genome (in)stability. Indeed, in addition to their well-known structural role, nuclear lamins seem to play several roles in DNA repair mechanisms. Accordingly, laminopathies such as HGPS are associated with genomic instability and defects in DNA repair and telomere maintenance. It has been also recently proposed that lamins play a role during replication or in the management of replicative stress.

### 5.1. Lamins and DNA Repair

HGPS patient fibroblasts and Prelamin A-expressing cells isolated from ZMPSTE24-deficient mice show increased levels of spontaneous DNA damages as visualized by the increased basal levels of γH2AX [251,280,281]. *LMNA*-deficient cells also show increased spontaneous γH2AX foci, chromosome/chromatid breaks, and aneuploidy, suggesting that A-type lamins could participate in DNA repair [282,283].

#### 5.1.1. Lamin A, Chromatin Modifications, and DNA Damage

Among the contributions of A-type lamins to DNA repair is their function in maintaining the positional stability of DNA repair foci, and their impact on damaged chromatin. For instance, a study revealed an association of Lamin A with chromatin via the histones H2AX and γH2AX [284]. These interactions were reported to increase after DNA damage due to the recruitment of damaged chromatin to A-type lamins [284]. Thus, A-type lamins may serve as anchors for DNA repair foci, thereby contributing to their positional stability.

It has been suggested that altered histone modifications in Progerin- or Prelamin A- accumulating cells participate in inefficient DDR and defective DNA repair. Several studies reported a decrease in the levels of the H3K9m3 heterochromatin histones mark in HGPS cells [230,250,285]. More recent reports precise that during early passages, HGPS cells harbor a high level of H3K9me3, which decreases with increased passages in parallel to Progerin accumulation [286,287]. In early passages, Prelamina- or Progerin-expressing cells display an increase in the methyltransferase SUV39H1, which is responsible of methylation of H3K9 [286]. It has been showed in HGPS that persistent DNA damage foci are associated with H3K9me3. Condensed chromatin might build up a barrier for the effective DNA repair. Knocking down SUV39H1 and SUV39H2 in HGPS or in *ZMPSTE24*−/− MEFs decreases H3K9me3, and persisting γH2AX and 53BP1 foci [286]. In contrast, at late passages, HGPS cells display reduced levels of H3K9me3 coupled with disrupted recruitment of SUV39H1 [287]. When in G0/G1, these cells demonstrate a defective amplification of the γH2AX signal mediated by a defective ATM activation in response to the DSBs, in addition to a defective recruitment of the 53BP1 and RIF1. Interestingly, in HGPS fibroblasts, the methylene blue, an antioxidant, which removes Progerin from the nuclear rim by increasing its solubility, restores the H3K9me3 level and rescues the defects in ATM activation, γH2AX signal amplification, and 53BP1 recruitment. Therefore, it is suggested that the loss of H3K9me3 could potentially impair ATM activation and consequently the downstream DDR upon DSBs in G0-G1 phase HGPS cells [287].

In addition, a study identified a role of Lamin A in regulating SIRT6-mediated functions in DNA repair [288]. SIRT6 is a stress-responsive deacetylase and mono-ADP-ribosylase enzyme. Its recruitment to damaged chromatin facilitates DDR signaling and DSB repair by recruiting the chromatin remodeler SNF2H, stabilizing DNA-PKcs at DSBs, and modifying CtIP and PARP1 [289]. Lamin A can directly interact with SIRT6 and facilitate its recruitment to damaged chromatin. Consequently, Lamin A could participate in SIRT6-dependent DNA-PKcs recruitment to chromatin, CtIP deacetylation, and PARP1 mono-ADP ribosylation in response to DNA damage, thereby suggesting that Lamin A regulates SIRT6-mediated DNA damage repair [288]. In HGPS, Progerin interacts more strongly to SIRT6 and tethers it to the nucleoskeleton. The functions of SIRT6 are then significantly impaired in response to DNA damages in HGPS fibroblasts [288]. Interestingly, deficiency of SIRT6 in mice lead to phenotypes that overlap with aging-associated degenerative processes [290]. In contrast, mice overexpressing SIRT6 exhibit an extended maximum lifespan [291].

**Figure 7:**
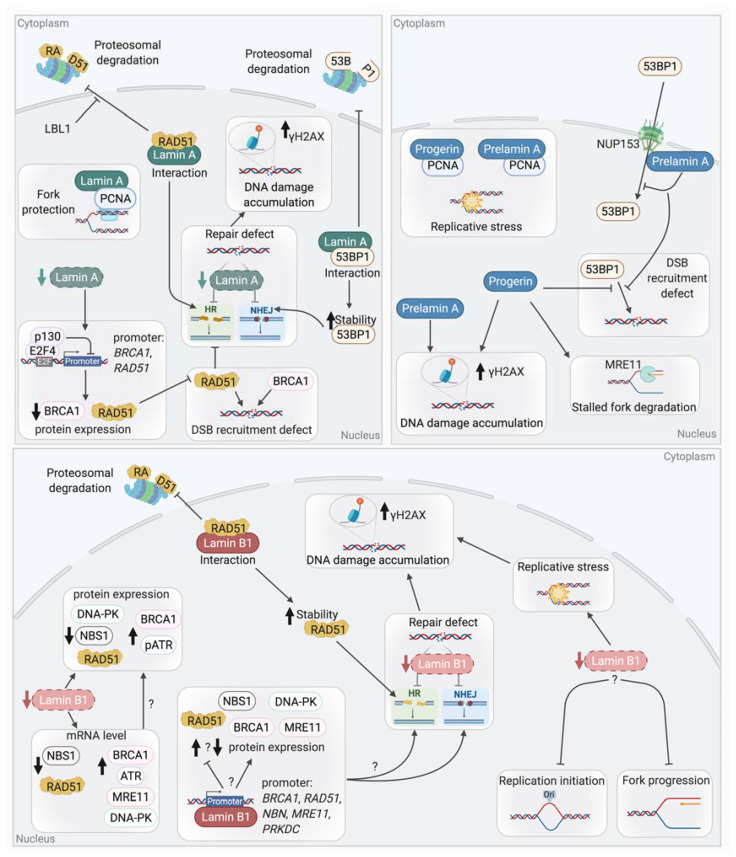
**Lamins deregulation leads to DSB repair defect and replication stress.** Upper left panel: Cell dysregulated for A type lamins show signs of genomic instability. Indeed, Lamin A has been shown to interact with RAD51, preventing its degradation by the proteasome. A similar interaction of Lamin A is noticeable with 53BP1, also increasing its stability. Decreased level of Lamin A reduces the expression of both RAD51 and BRCA1 through E2F4/p130-dependent repression of their promoter. Their defective recruitment at DSB leads to a decreased HR and NHEJ efficiency. Lamin A also participates to RF stability by its interaction with PCNA. Upper right panel: Cells accumulating Progerin or Prelamin A show DNA damage accumulation (increased level of spontaneous γH2AX foci) and a defect of 53BP1 recruitment to DSB. Indeed, Prelamin A accumulation impairs 53BP1 nuclear import by NUP153. Progerin and Prelamin A both interact with PCNA, sequestrating it away from the RF. In Progerin expressing cells, the defective recruitment of RAD51 at stalled fork leaves them unprotected and subject to extensive degradation by MRE11. Taken together, these observations explain in part the reported replication stress in HGPS cells. Bottom panel: Lamins B1 plays a role in DNA repair mechanism and its loss induces DNA damage accumulation. Indeed, as with Lamin A, Lamin B1 interacts and stabilizes RAD51, thus decreased level of Lamin B1 leads to DSB repair defect. In addition, diminution of Lamin B1 leads to a dysregulation of mRNA and proteins levels of key repair factor such has an upregulation of BRCA1, ATR, and a decrease of DNA-PK, RAD51 and NBS1 protein levels. A role of Lamin B1 in replication has also been reported. Lamin B1 extinction induce replicative stress and an extension of the S phase duration, which suggest a role of Lamin B1 in late origin firing and/or fork progression.

Links between lamins and the mechanisms of DSB repair, NHEJ, and HR, have been reported (Figure 7). Cells with mutated or deficient Lamin A/C showed an increase in radio-sensitivity and in DNA breaks as revealed by the persistence of γH2AX foci. These results suggest that DSB repair could be defective in these cells [282,283,292,293].

#### 5.1.2. Lamin A and NHEJ

Studies have shown that A-type lamins participate in the repair of DSBs through the regulation of NHEJ. For instance, *LMNA*-deficient MEFs show a defective long-range NHEJ [283]. Comet assays revealed defects in the fast phase of repair of IR-induced DSBs, suggesting a defect in NHEJ in these MEFs [292]. This phenotype is partially explained by the loss of 53BP1, a key factor of NHEJ. An interaction has been reported between Lamin A/C and 53BP1 in basal conditions where Lamin A/C shield 53BP1 from UbxH7-dependent proteasomal degradation [282,294]. Thus, LMNA-depleted cells show an impaired formation of 53BP1 foci after irradiation due to a decreased stability of 53BP1 and a reduction in its protein levels.

HGPS and *ZMPSTE24*-deficient cells show defects in DSB repair mechanisms. These defects are attributed, at least partially, to alterations in the expression and recruitment of several DSB repair factors to DNA damage sites due to the presence of Progerin or Prelamin A. 

Indeed, as discuss above, altered histones modifications or chromatin compaction may explain defect of DNA repair factors recruitment. Recently, it has also been shown that Prelamin A accumulation impairs 53BP1 recruitment due to a defective nuclear import of 53BP1 by NUP153 [295]. Similarly, HGPS patient cells present a decrease in 53BP1 foci formation and a delay in the colocalization of 53BP1 with γH2AX.

These results show that the level of Lamin A has to be finely regulated to avoid defects in 53BP1 recruitment and NHEJ-dependent DSB repair.

#### 5.1.3. Lamin A and Homologous Recombination

Lamin A may also play a role in HR-dependent DSB repair. Indeed, it has been reported that *LMNA*-deficient cells showed reduced HR efficiency [292]. However, a different study showed that the HR efficiency was not altered upon LMNA depletion (even though the same cell line with the same HR reporter construct were used) [296]. Different expressions of the endonuclease I-SceI used here to induce break in the reporter, or different Lamin A level extinction between both studies might explain the different results observed. However, a decrease in RAD51 expression, associated with a defect in RAD51 foci formation upon irradiation, was reported upon Lamin A depletion, suggesting a defect of HR. Similar results were obtained for BRCA1 [292].

The observed defects of RAD51 and BRCA1 appeared to be due to the transcriptional repression of these genes by the Rb family member p130, which forms a transcriptionally repressive complex with E2F4. Co-IP experiments showed that the depletion of Lamin A promotes an increased formation of p130/E2F4 complexes [296]. It is important to note here that in contrast to what was observed upon the depletion of Lamin A using shRNA in MCF7 or MEFs, unaffected levels of RAD51 were observed in *LMNA* mutant (Y259X) HDFs isolated from a patient [282].

Interestingly, HGPS cells present a defect in RAD51 and BRCA1 recruitment [280]. Progerin accumulation also induces alterations in DSB repair by impacting PARP1. Indeed, depleted levels of PARP1 lead to accumulation of SSBs, which are converted to DSBs during replication. These effects were due to a disrupted nuclear import of PARP1 caused by the abnormal anchorage of Progerin to the NE [297].

#### 5.1.4. Lamin B1 and DSB Repair

Lamin B1 also plays a role in DSB repair (Figure 7). Indeed, the depletion of Lamin B1 in U2OS and HCT116 cancer cell lines leads to chromosomal instability and persistent DNA damages. These damages were revealed by the numerous spontaneous γH2AX and 53BP1 foci, indicating an accumulation of DSBs [298]. Indeed, the analysis of HR and NHEJ efficiencies in these cells showed that both pathways are less efficient, thus explaining the accumulation of DSBs. Alterations in the mRNA and protein levels of several major DSB repair proteins are observed upon Lamin B1 depletion. For instance, increases in 53BP1, BRCA1, ATR, RAD50, and MRE11 levels are seen in Lamin B1-depleted cells. On the contrary, the protein level of DNA-PKcs, NBS1, and RAD51 were dramatically reduced in these cells. It was also assumed that improper assembly of the MRN complex takes place in these cells due to the reduction in NBS1 levels [298]. Although the misregulation of some DSB repair factors could explain the accumulation of DNA damages, supplementary investigations are still required to associate the reduced levels of these factors with the observed defects in NHEJ or HR. Finally, another study also identified a role of Lamin B1 in the control of HR via its interaction with RAD51 and the stabilization of this key HR factor [299]. The transient depletion of Lamin B1 by siRNA in U2OS cells led to an increased sensitivity to IR and an impaired HR efficiency. In these cells, the formation of RAD51 foci after IR was impaired. It was proposed that an interaction between Lamin B1 and RAD51 favors the stabilization of RAD51 after IR, through the inhibition of proteasomal degradation, thereby regulating HR [299].

#### 5.1.5. Lamins and Other DNA Repair Mechanisms

Recently, the role of lamins in other DNA repair mechanisms such as Base excision repair (BER) or Nucleotide excision repair (NER) has been also investigated. Indeed, *LMNA*-null cells showed a reduced expression of proteins involved in BER such as PARP1, LIG3, and POLβ and a decreased activity of APE1 and POLβ. These defects led to an accumulation of 8-oxoguanine and an increased frequency of substitution mutations in *LMNA*-deficient cells [300].

Another study reported that HGPS patient cells and cells deficient for ZMPSTE24 show an aberrant accumulation of the XPA protein, which is involved in NER, at sites of DNA damages [301]. 

It has been also shown that a decrease in Lamin B1 levels leads to a defect in UV-induced DNA damage repair, associated with an increased sensitivity to UV irradiation, a delay in DNA repair foci formation, and in damage removal. These results are correlated with a reduction in the protein levels of DDB1 and CSB, both of which are key factors of NER [302].

Altogether, these studies suggest that the levels of lamin proteins have to be finely regulated to avoid defects in expression, stability, or nuclear transport of different key DNA repair factors, in addition to subsequent DNA repair defects and genome instability.

### 5.2. Lamins and Telomere Maintenance

In mammals, the protective T-loop structure of telomeres and the complex of telomeric proteins, named shelterin, found at telomeres, are essential for genome stability [303,304]. Indeed, this particular structure and the associated proteins ensure that the telomere extremities are not recognized as DSBs, thus preventing an ATM-dependent DDR and NHEJ or HR to occur at the ends of chromosomes. A defect in telomere protection induced by the functional loss of one of the major shelterin proteins, TRF2 (by the expression of TRF2ΔBM), leads to damages at telomeres (known as telomere dysfunction-induced foci, TIFs), end-to-end chromosome fusions, senescence, or apoptosis [305,306,307,308]. In addition to its role in T-loop formation [309,310,311,312], TRF2 inhibits the ATM-dependent DNA damage response as well as NHEJ and HR at telomeres, thanks to its interactions with different factors involved in these processes [313,314,315,316,317].

Links between telomeres, NE and lamins, especially Lamin A/C, have been reported. In mammals, meiotic telomeres are clustered to the NE and move along during meiotic prophase I [318]. After mitosis in somatic cells, at the stage of NE reassembly, telomeres are transiently enriched at the nuclear periphery [319]. Recently, contact between telomeres and the nuclear rim was also detected using the MadID approach in G1/S arrested HeLa cells [320]. A subset of telomeres is also found close to the nuclear periphery during replication [321]. As previous studies had suggested that human telomeres are attached to the nuclear matrix [322], lamins may play a role in tethering telomeres to the nuclear matrix or to the NE, (most) likely in different phases of the cell cycle.

During the senescence of mesenchymal stem cells, an aggregation and re-localization of telomeres at the lamina were observed [323]. Interestingly, a shortening of telomeres has been reported in HGPS cells [248,324,325] and also upon the expression of Progerin in fibroblasts [326]. This shortening is associated with TIFs and telomeric aberrations. Recently, it was proposed that the DDR activation at telomeres participates in progeroid detrimental phenotypes of HGPS mouse model [327]. In addition, a deficiency of Lamin A in mouse cells leads to the attrition of telomeres and a defect in their localization. Moreover, an interaction between Lamin A/C (but not Progerin) and TRF2 stabilizes T-loops with interstitial telomeric sequences (ITL), suggested/proposed to be novel chromosome-end structures [328]. 

The impact of Lamin B1 on telomere stability has been poorly explored. Our team showed that Lamin B1 overexpression leads to NE alteration and induction of senescence [239]. Interestingly, another team reported that this proliferative defect in Lamin B1-overexpressing cells is rescued by the telomerase catalytic subunit hTERT, suggesting that telomeric alterations could be involved in this phenotype [263]. 

Altogether, these data suggest that the levels of lamins or the NE/matrix organization play an important role in DNA repair mechanisms and telomere maintenance. Thus, one could suggest that a misregulation of lamins may participate in genome instability, aging phenotypes, and may also participate in tumorigenesis, particularly in the case of senescence escape.

### 5.3. Lamins, Replication, and Replicative Stress

Several lines of evidence also reveal an implication of lamins in DNA replication and in the management of replicative stress (Figure 7).

#### 5.3.1. On Replication Progression

Early studies in *Xenopus* eggs extracts depleted for lamins or expressing mutant form of lamins agree on the necessity of an intact NL for proper replication and PCNA localization [329,330,331,332]. However, these studies implicate lamins either in the initiation phase of replication [329,330] or in its elongation phase [331,332]. Lamin A and Lamin B1 nuclear foci/structures are also found at replication sites, respectively, in early or mid-late S phase (PCNA staining colocalization or BrdU incorporation) [218,333]. Consistently, Lamin A is associated in the early S phase with MCM3 and the replicative polymerase POLε, POLα, and POLδ, while in the late S phase, only the association with MCM3 and POLε remains [334]. More recently, a lamins IgG-like domain was shown to interact with PCNA in vitro [335]. The PCNA-Lamin A interaction was also confirmed by GST-pull down in non-challenged cells [336] and in a yeast two-hybrids screen [337]. Interestingly, Prelamin A and Progerin seem to interact even better with PCNA that mature Lamin A [336,338,339,340]. Those observations have led to the hypothesis that Prelamin A and/or Progerin sequesters PCNA, as evidenced by the diminution of MCM7-PCNA interaction in HGPS cells [339] or by the increased PCNA monoubiquitination and the increased colocalization of POLη and PCNA foci with γH2AX after Prelamin A ectopic expression [336]. PCNA interaction with Prelamin A and/or Progerin would therefore prevent its interaction with Lamin A and induce stalled RF, thus revealing a role for Lamin A in RF progression.

Interestingly, more mechanistic insight has emerged in recent years concerning Lamin A implication in the replication process although the picture is still incomplete. *LMNA*^−/−^ cells spend more time in the S-phase [341] while Progerin expressing cells have delayed passage through the S-phase [340]. Importantly, BrdU CHIPs analyses and iPOND results reveal the presence of Lamin A (and C) on newly synthetized DNA along with PCNA [298,340], but not that of Progerin [340]. Besides, while less PCNA immunoprecipitated from Progerin expressing cells, γH2AX is found on the nascent DNA in these cells, reinforcing the idea that Progerin sequesters PCNA, away from the active RF. Thus, PCNA sequestration could be one reason behind the replicative stress observed [340]. Moreover, it was recently observed that while Lamin A overexpression had no effect, ectopic expression of progerin in unchallenged cells induces slowdown of the RF progression and increases its stalling, leaving the fork unprotected and subjected to MRE11-dependent degradation [342].

Reports of a role of Lamin B1 in replication have also been made. Lamin B1 extinction seems to delay growth due to the extension of the S phase duration, rather than a defect of S-phase entry and suggest the inhibition of late origin firing and/or of fork progression [343]. Indeed, Lamin B1-depleted cells are capable of EdU incorporation, despite spending more time in the S phase and present signs of replicative stress [343]. Although Lamin B1 was not associated with newly synthesized DNA, less PCNA and RPA were bound to the nascent DNA in Lamin B1-depleted cells [298]. The increase in CDC6 protein level, the sensitivity to depletion of this licensing factor in LMNB1-depleted cells [343], and the observations mentioned above leads to the idea that Lamin B1 could have a role in the replication initiation, such as assembly of replicative complex and in fork progression, especially during the elongation phase.

#### 5.3.2. Replication Timing

In eukaryotic cells, large regions of chromosome replicate simultaneously and in a temporally ordered manner, a phenomenon called the replication timing (RT) program [344].

The replication timing is very robust, cell-type specific, and more or less conserved between close species, although differences have been reported during differentiation. Genome-wide replication timing studies have more precisely revealed the existence of megabases domains of chromosome that replicate at defined time during the S phase and called constant timing regions (CTR). In-between early and late CTR, timing transition regions (TTR) have progressive changes in replication timing. Correlations have been made between transcription and replication timing. Indeed, actively transcribed region tends to replicate earlier in the S-phase, but differences can occur depending of the developmental stage or cell types. Interestingly, Lamina-associated domain (LAD) often have repressed chromatin and tend to have late replication timing [345,346]. The LADs have also been mostly mapped to late CTR and TTR. These genomic studies are also supported by the replication timing analysis of tagged loci in DT40 cells, where late replicating loci tend to be in closer proximity to Lamin B1. Furthermore, the allelic asynchrony in replication timing at the same locus is higher when the first allele to replicate is located in a central compartment and the second one at the periphery, as compared to both allele of the same locus being at the periphery of the nucleus [347]. Among the factors identified as playing a part in replication timing, RIF1 seems to be a primordial one. In the mouse genome, RIF1 interacting domains mostly correspond to late replicating regions and therefore strongly coincide with LAD. Of note, RIF1 is found at the nuclear periphery and coimmunoprecipitates with Lamin B1. Interestingly, as Lamin B1 interacting domains, not bound to RIF1, have less synchronized replication timing and cannot be strictly categorized as a late replicating region, RIF1 seems essential for late replication only in regions devoid of Lamin B1 interactions [348]. Therefore, one could speculate that Lamin B1 through interaction with RIF1 might participate in the synchronized (late) replication of interacting domain. The role of lamins in replication timing was emphasized by a recent study showing that temporal order of replication is altered in Progeria (HGPs) cells. A specific “Progeria” RT signature, containing regions that replicate early in progeria, but replicate later in cells from healthy donors, was identified. Interestingly, one of the earliest RT alterations corresponds to the *P63* gene. This RT defect was also found in fibroblasts from Rothmund-Thompson syndrome patients, another premature aging disease. The authors suggest that this altered RT and the subsequent abnormal *P63* gene expression could be linked to some pathophysiological manifestations of progeroid diseases [349].

#### 5.3.3. During Replicative Stress

Lamins also seems to intervene in the management of the replicative stress, essentially through defective recruitment of DNA repair or stalled fork protection factors.

Lamin A was reported to maintain the stalled RFs [296]. Indeed, LMNA-depleted cells have increased sensitivity to treatment preventing RF progression such as topoisomerase inhibitors (camptothecin), crosslink inducing agents (cisplatin, mitomycin C, formaldehyde) or replication stress inducing agents (hydroxyurea, HU). Interestingly, release from HU increased stalled fork and reduced new origin firing in these cells, while more radial chromosomes are observed. Surprisingly, no HR repair efficiency defect was measurable in these cells, although defective recruitment of repair factor (such as MRE11, CtIP, RAD51, RPA, FANCD2) was found after the HU treatment [296]. Yet, in another study, although the monitoring of HR repair efficiency was performed with the same reporter, a defect was reported after shLamin A depletion and correlated to a decrease protein and transcription level of BRCA1 and RAD51 [292]. The difference in lamin A downregulation level might contribute to the difference observed in these HR repair efficiency reports.

Using a Lamin-binding ligand (LBL1), a Lamin A-RAD51 interaction was identified to prevent RAD51 degradation or to ensure RAD51 stability and might therefore protect HR repair efficiency [350]. These reports suggest that the defective HR repair or stalled fork protection might be the issue behind replication stress management.

In Progerin-accumulating cells, the replication stress and damage observed is proposed to result from the sequestration of PCNA by Progerin, leading to an aberrant recruitment of XPA at stalled/collapse forks and the subsequent defective recruitment of repair factors, such as RAD50 and RAD51 [301,339].

Therefore, it seems there is a bundle of evidence suggesting that Lamin A deregulation impairs RAD51 function in replicative stress management.

Lamin B1 seems also involved in replication stress management. Indeed, LMNB1 depletion increases fork stalling and impedes fork restart upon release from HU treatment [298]. The LMNB1-depleted cells also affected levels of repair proteins such as an increase in 53BP1, BRCA1, pATR, CHK1, and pCHK1 but a decrease in NBS, RAD51, and DNA-PK. Following camptothecin treatment, LMNB1-depleted cells presents decreased survival, which is also accompanied by the absence of MRE11 recruitment. This suggest that Lamin B1 defect could affect the recruitment of protein implicated in early step of the DDR, such as signalization through the recruitment of MRN complex. Lamin B1 also binds the promoter of many HR proteins, leaving the possibility that it might regulate their expression, although no absolute correlation has been made so far. For instance, Lamin B1 binds to both *RAD51* and *BRCA1* promoter and respectively a decrease and an increase level of the protein is found in LMNB1-depleted cells [298].

## 6. A Link between Nuclear Envelope Integrity, DNA Damage, Inflammation, and Aging: The Case of the HGP Syndrome

The mechanisms leading to “inflammaging” in premature pathological aging or in physiological aging must be precisely understood in order to be able to improve health during old age. Different reports suggest a link between nuclear organization or NE integrity, lamins, inflammation, and aging [351,352]. Indeed, NE and/or micronuclei integrity are important to shield genetic material against genotoxic insults (such as oxidative stress as discussed in [259]) and importantly to avoid leakage of self-DNA in cytoplasm, which will trigger an inflammatory response, being recognized by different DNA sensors such as cGAS protein. It has been shown that the cGAS-STING signalling is activated in HGPS cells [342] and in a murine progeria model [353] (likely due to DNA repair defect or replicative stress). The AIM2 inflammasome pathway is also activated by pharmacological disruption of NE integrity [354].

Here we discuss the link between replicative stress, NE integrity, and inflammation in the case of HGPs.

**Figure 8:**
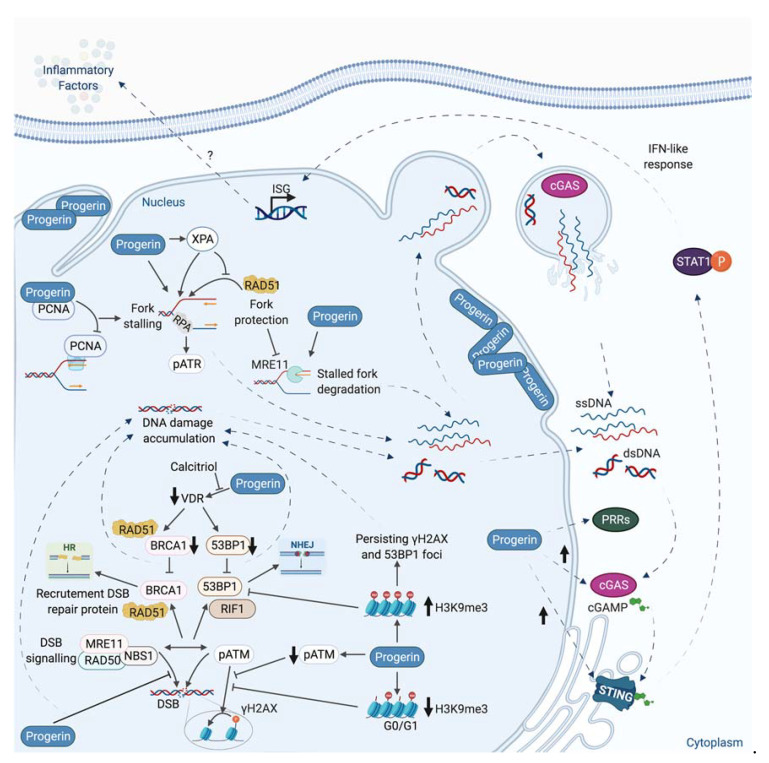
**Progerin expression in HGPS leads to DNA repair defect, replicative stress, and inflammation through DNA sensing in the cytosol.** Expression of Progerin and the ensuing genomic instability has recently been reported to contribute to the activation cGAS-STING pathway and the production of an inflammatory response that may accelerate the aging process in HGPS cells. Indeed, Progerin accumulation leads to replicative stress by the sequestration of PCNA, away from the fork, and aberrant recruitment of XPA to stalled fork, which impedes their RAD51-dependent protection leading to degradation of stalled fork by MRE11. HGPS cells present an important accumulation of DNA damage due in part to the decreased level of VDR caused by Progerin which leads to a diminution of key DNA repair factor such as RAD51, BRCA1 (leading likely an HR defect) and 53BP1. Recently, it was reported that a calcitriol treatment could reduce the effect of Progerin accumulation on the VDR level, thus partially rescuing DSB repair mechanism in HGPS cells. It has also been shown altered histone modifications in Progerin accumulating cells, leading to reduced level of H3K9me3 in G0/G1 cells, inducing a defective ATM activation and defective amplification of the γH2AX signal in response to the DSBs and an impaired recruitment of 53BP1 and RIF1 during NHEJ. In contrast, it has been also reported (in early passage), an increase of SUV39 protein and H3K9me3; the condensed chromatin preventing access to DNA repair factors lead to persistent DNA damage. Depletion of SUV39 H1 rescue the DNA repair factor recruitment. As described previously, replicative stress and DNA damage accumulation (through the different mechanisms described here) can lead to cytosolic DNA production, which activate the cGAS-STING pathway and production of inflammatory factors. Moreover, Progerin accumulation has been reported to increase proteins levels of cGAS, STING, and PRRs and induce a IFN-like response mediated by STAT1. As a result, Progerin cells show an increased level of ISG.

Recent data support that replication stress could be a major cause of genomic instability in laminopathies/or as observed in HGPS, which could contribute to the activation of an innate immune responses to self-DNA and inflammation that in turn may accelerate the aging process (Figure 8).

Recently, the team of Gonzalo showed an increase in inflammatory cytokine expression through cGAS activation upon Progerin expression [8,342]. This result is in agreement with the previous description of a NF-KB- dependent inflammation observed in *ZMPSTE24*^−/−^ mice (a murine progeria model) [252], since NF-KB activation may result also from cGAS-STING-TBK1 pathway. Importantly the cytokine expression was linked to replicative stress observed in HGPS. Indeed, fiber assay show an augmentation of stalled RF upon Progerin expression. In addition, nucleolytic degradation of stalled RF was observed upon Progerin expression. This could be explained by deprotection of stalled forks, due for instance to a defect of RAD51 and BRCA1 recruitment in HGPS cells. Indeed, Mirin treatment, an MRE11 inhibitor, rescued fork degradation, showing an involvement o MRE11 [342]. An uncontrolled degradation by nucleases of RFs was previously linked with cytosolic DNA production and cGAS-STING activation in context of SAMHD1 deficiency [5,212]. In addition, *BRCA1*^−/−^ defective cells also show an increase of cytosolic DNA. 

Importantly, cytokine expression after Progerin expression is abolished after calcitriol (Vitamin D) treatment. Indeed, Vitamin D receptor (VDR) is involved in genome stability, and HGPS cells reduce the expression of VDR due to Progerin accumulation [97]. Treatment with calcitriol increases VDR level and restores RAD51 levels. Calcitriol treatment was also comparable to the mirin treatment and rescue decreased stalled fork degradation in Progerin expressing cells, thus leading to a diminution of IFN response [342].

Expression of Progerin has been reported in atherosclerotic coronary arteries from aging individuals [233,355]. Interestingly, Progerin overexpression in endothelial cells recapitulates some features of aging-associated endothelial cell dysfunction, including a proinflammatory phenotype and oxidative stress, together with persistent DNA damage. In accordance with a pathogenic role for the persistence of the farnesyl moiety of Progerin, pharmacological inhibition of farnesylation partly restored endothelial cell function [254].

In combination with the genetic instability and replicative stress observed in Progerin expression, it is possible that the perturbation of NE structure in HGPS cells could also favor genomic DNA leakage and its release into cytoplasm. Cytosolic DNA accumulation activates the cGAS-STING pathway and production of inflammatory factors [356]. Replicative stress and DNA damage lead, upon mitosis, to micronuclei formation, shielding mis-segregated chromatin or lagging chromosomes. Micronuclei envelope fragility has been linked to cGAS activation during mitosis [357]. Indeed, cGAS has been shown to colocalize with micronuclei and the sensing of DNA into micronuclei could activate the STING pathway [358]. Recently, cytoplasmic chromatin fragments (CCF) present in the micronuclei derived from the main nucleus were shown to trigger cGAS-STING innate immune signaling and cellular senescence [359].

In addition, with NE fragility, the disruption of micronuclei envelope may lead to DNA leakage and cytosolic DNA accumulation in HGPS. It has been shown that Lamin B1 depletion, but not Lamin B2 and A/C, leads to micronuclei disruption, which was characterized by large holes in the micronuclei envelope. Interestingly overexpression of Lamin B2 impedes this mechanism, likely by avoiding rupture of the micronuclei envelope [360]. The importance of lamins in micronuclei stability and in subsequent cellular responses has been illustrated recently by a study showing that, upon paclitaxel treatment, cGAS co-localizes with micronuclei and triggers not only an inflammatory response but also apoptotic death of neighbour cells. This cellular death is dependent of the pro-apoptotic Bcl-2 family member, NOXA. Interestingly overexpression of Lamin B2 impedes this mechanism, likely by avoiding rupture of the micronuclei envelope [361].

In agreement, with this hypothesis linking NSA, micronuclei, and inflammation, it has been recently reported that the Muscle stem cells (MSCs) from *ZMPSTE24*^−/−^ mice, exhibit increased nuclear abnormalities, cytoskeletal stiffness, and the presence of cytoplasmic chromatin fragment in cGAS-positive micronuclei. These cells have elevated expression of SASP that negatively impact muscle stem cell function. Interestingly, RhoA signalling promotes nuclear abnormalities and cellular senescence in these cells. Inhibition of this signalling reduces F-actin polymerisation, nuclear blebbing, DNA damage, cGAS-STING signaling, and cellular senescence. Consequently, the RhoA inhibition rescue extended the defective phenotypes in skeletal muscle and extended the healthspan of *ZMPSTE24*^−/−^ mice [353]. This study suggests that abnormal activation of cGAS-STING pathway could be detrimental in HGPS and that RhoA might serve as a promising target for therapeutic treatment of HGPS. Rescue of NE shape alteration was also previously proposed as a therapeutical strategy for HGPs. This rescue of nuclear shape can be performed by Remodelin, an NAT10 acetyl-transferase inhibitor [362,363]. Interestingly, it was recently published that NAT10 may be involved in micronuclei formation (through regulation of DNA replication) and may have activated a SASP signaling in colorectal cancer cells [364].

Finally, one could also propose that Progerin expression may lead to inflammation, by affecting chromosome domain organization, especially chromatin location at the periphery and alteration of gene expression associated with LADs and consequent abnormal induction of some inflammatory factors as SASP genes, as has been observed in the case of Lamin B1 extinction [261].

## 7. Concluding Remarks

In this review, we present the DSB repair mechanisms, the important role of HR factors to protect and restart stalled forks. The in-depth knowledge of DNA repair mechanisms and especially of management of replicative stress has already made it possible to propose novel anti-tumoral strategies. Using the concept of synthetic lethality, targeted treatment with PARP1 inhibitors of HR-deficient tumors is one example [365,366,367,368,369]. However, the emergence of resistance to this treatment still requires active research in this area of DSB repair to continue improving our knowledge and offer personalized treatment.

Studying the link between DNA damage accumulation, replicative stress, and cGAS-dependent inflammation is also important for understanding many human pathologies. These pathologies include inflammatory pathologies (such as Aicardie Gouttières), severe inflammatory situations (such as in the event of a “cytokine storm” during viral Covid19 infection), cancers and also pathologies associated with normal or premature aging. As also proposed by others, targeting the cytoplasmic chromatin-mediated pathway may hold promise in the treatment of inflammation-related disorders [359]. In addition, a link between STING activation and efficiency of radiotherapy [370,371,372] and also immunotherapy [373,374] has recently been highlighted. Novel strategies are proposed to strengthen the STING pathway, in combination (or not) to immunotherapy, to improve conventional anti-tumoral treatment, especially to transform cold tumor (refractory to immunotherapy) to hot tumors.

We also discuss the role of lamins in DNA repair. Altered level lamins affect the recruitment of key DSB repair (and replication) factors, through different mechanisms (altered transport through nucleus, gene expression and/or DNA damage signaling). This raises the question about their potential role more generally on genome stability and their potential role during tumorigenesis. Alteration of NE shape has been used for decades by cytologists as a prognostic factor for tumor aggressiveness. However, the causes and consequences on the tumorigenesis process are still unclear. Of course, misregulation or altered levels of lamins found in different tumors may account for the NE shape alterations. Misregulation of lamins are often associated with tumor aggressiveness and metastasis, and this alteration of lamin level, especially increased level of Lamin B1, has been suggested as a biomarker and even as a therapeutical target [271]. How the lamins level is altered is not yet understood in different tumors. It could be suggested that oxidative stress, which was also associated with aggressive stage of tumors and potentiate tumor dissemination [375,376], might participate in the increase of Lamin B1 level. Indeed, our team and others showed that Lamin B1 is upregulated in oxidative stress situations [239,240,259,265]. Since it has not been reported yet that mouse models with lamins deficiency or lamins overexpression are more prone to develop cancer, it is unlikely that alteration lamins level participate alone in the initiation of tumorigenesis. However, recent studies highlight the role of lamins in DNA repair and in replication, consequently in the control of inflammatory factors production. This might suggest an impact of the dysregulation of these key nuclear components in tumorigenesis progression through genome instability and the production of an inflammatory tumor environment, which are both important hallmarks of cancer [277]. In addition, deformation or fragility of NE can also lead to DNA damage (as it has been shown during constrained cellular migration [377]), and could therefore participate in a leakage of DNA fragments in cytoplasmic compartment or their increased accessibility by DNA sensors. Although the innate immune response is beneficial in destroying cells carrying DNA damages and may participate in protection against tumor development, the accumulation of DNA damage may lead to a chronic inflammatory microenvironment, which could instead favor tumor progression. For instance, IL-6 or IL-8 production is associated with the epithelial-mesenchymal transition [378]. The altered level of lamins and thus the alterations of the NE may also have an impact on the formation of micronuclei and on their permeability (to the cGAS factor for example). The formation of micronuclei upon mitosis is a consequence of genome instability (consequence of DNA repair defect or replicative stress), but could be also a cause of alterations in the genome since it has been suggested to participate in chromothrypsis [357,379,380].

The level of Lamin B1 is also affected during the establishment of senescence and also in different tumors. Decrease of Lamin B1 affects the expression of many DNA repair factors, although the cause of these differential expressions is not fully understood. However, to know whether lower and higher level of lamins could directly participate in or worsen the genome instability and thus participate in tumorigenesis, especially after escape of senescence, require further investigations.

Another important role of lamins is their involvement in senescence and aging. What can we learn about the recently described connection between NE envelope alteration, genome stability, and inflammation?

It is known that Progerin is expressed during normal aging [230,231,232,233,234]. Expression of Progerin leads to DNA damage accumulation (as it is observed in HGPs cells) and also participates in replicative stress and inflammation through activation of the cGAS-STING pathway. Further experiments remain to be done to address the role of Progerin in inflammatory pathologies associated with normal aging. The mechanisms leading to “inflammaging” in premature pathological aging or in physiological aging must be precisely understood in order to consider (therapeutical approaches) an improvement of health during old age. Important novel connection between cGAS, senescence, SASP and aging has been reported [6,213,216,381,382]. Targeting the pathway(s) responsible for the formation/signalling of cytosolic DNA may offer a new therapeutical avenue for treating diseases relating to normal or premature aging.

In conclusion, recent findings and current research establishing, on the one hand, the link between DNA damage, in particular replicative stress with inflammation, and on the other hand, the relationship between the integrity of the NE and the response to DNA damage, will undoubtedly bring progress in the understanding of the mechanisms of premature or normal aging and also of tumorigenesis. Ultimately, this will bring therapeutic avenues to improve health during old age, which is a major societal issue, but also to new personalized anti-tumor strategies.

## Data Availability

Not applicable.
